# *Familias con Orgullo*: Study protocol for an efficacy study of a family-based intervention for Hispanic sexual minority youth

**DOI:** 10.1371/journal.pone.0295683

**Published:** 2023-12-15

**Authors:** Alyssa Lozano, Yannine Estrada, Dalton Scott, Maria I. Tapia, Hudson P. Santos Jr., Adam W. Carrico, Joseph Zolobczuk, Amber Manker, Guillermo Prado

**Affiliations:** 1 School of Nursing and Health Studies, University of Miami, Coral Gables, Florida, United States of America; 2 Department of Health Promotion and Disease Prevention, Robert Stempel College of Public Health & Social Work, Florida International University, Miami, Florida, United States of America; 3 YES Institute, Miami, Florida, United States of America; 4 Alliance for LGBTQ Youth, Miami, Florida, United States of America; PLOS: Public Library of Science, UNITED KINGDOM

## Abstract

This manuscript describes the rationale and design of a family-based, Hispanic sexual minority youth (HSMY) specific preventive intervention, *Familias con Orgullo* (Families with Pride). HSMY (*N* = 306) and their primary caregivers will be recruited in South Florida and be randomized to Familias con Orgullo or prevention as usual. The intervention will be delivered by trained study facilitators. Outcomes will be measured at baseline and 6-, 18-, and 30-months post-baseline. The goals of this study are to evaluate whether the Familias con Orgullo intervention, compared to community practice, is effective in reducing drug use and depressive symptoms through the improvement of parent support for the youth, parent acceptance, family functioning, youth stress, and sexual minority stress. Additionally, we will explore whether gender and baseline levels of parent support for the youth, parent acceptance, family functioning, youth stress, and sexual minority stress moderate intervention effects on the youth outcomes.

**ClinicalTrials.gov identifier:** NCT06057337, First posted September 28, 2023.

## Introduction

Hispanic sexual minority youth (HSMY) face a disproportionate burden of drug use and depressive symptoms [1–3] relative to both Hispanic non-sexual minority peers [4, 5] and non-Hispanic White sexual minority youth [6]. Despite the pervasive health disparities for HSMY, there are limited evidence-based interventions available for the prevention/reduction of drug use and depressive symptoms [7, 8]. Moreover, the limited interventions that are available do not include the family and are not culturally-syntonic, meaning that they do not address Hispanic cultural values and beliefs [7, 8]. Including these two components is particularly important for HSMY because in many cases, Hispanic families may be less accepting of their youth’s sexual orientation due to cultural values and beliefs [9] thereby increasing risks for drug use and worsening health related outcomes for HSMY [10, 11].

Given the centrality of family for HSMY and the possible impact family may have on health outcomes, studying risk processes only at an individual-level may not capture other simultaneously operating risk processes (e.g., parent support for the adolescent, parent acceptance, family functioning). Important family level factors suggest that there is a need for family-based interventions to address the health disparities HSMY face as it relates to drug use and depressive symptoms. Extensive research has documented the efficacy and effectiveness of family-based interventions for the prevention and/or reduction of drug use and depressive symptoms [12, 13], however, these interventions were not originally developed for HSMY and do not address the unique needs of this population [14]. Despite the evidence that family-based interventions can improve health outcomes for Hispanic youth and the importance of highlighting and supporting family influences on sexual minority health [15, 16], there is a dearth of evidence-based, culturally congruent family interventions that target the multiple risk and protective factors relevant to HSMY and their families to ameliorate health disparities related to drug use and depressive symptoms. Therefore, the purpose of this manuscript is to describe the protocol for a randomized controlled trial to evaluate the efficacy of Familias con Orgullo.

To evaluate the efficacy of Familias con Orgullo in preventing drug use and depressive symptoms, this study will use a randomized trial design with two arms (Familias con Orgullo and community practice; see study design and intervention conditions below). We hypothesize that Familias con Orgullo will be efficacious, compared to community practice, in decreasing past 90-day drug use frequency and quantity and depressive symptoms among HSMY, over 30-months and that the intervention effects on youth outcomes will be partially mediated by changes in parent support for the youth, parent acceptance, family functioning, youth stress, and sexual minority stress. In addition to our main hypothesis, this study also aims to explore whether gender and baseline levels of parent support for the youth, parent acceptance, family functioning, youth stress, and sexual minority stress moderate intervention effects on the youth outcomes. We hypothesize that gender and baseline levels of parent support for the youth, parent acceptance, family functioning, youth stress, and sexual minority stress will moderate intervention effects on youth outcomes such that youth at higher risk (e.g., families reporting higher stress, lower support, and poorer family functioning) will benefit most from Familias con Orgullo.

## Materials and methods

**Fig 1:**
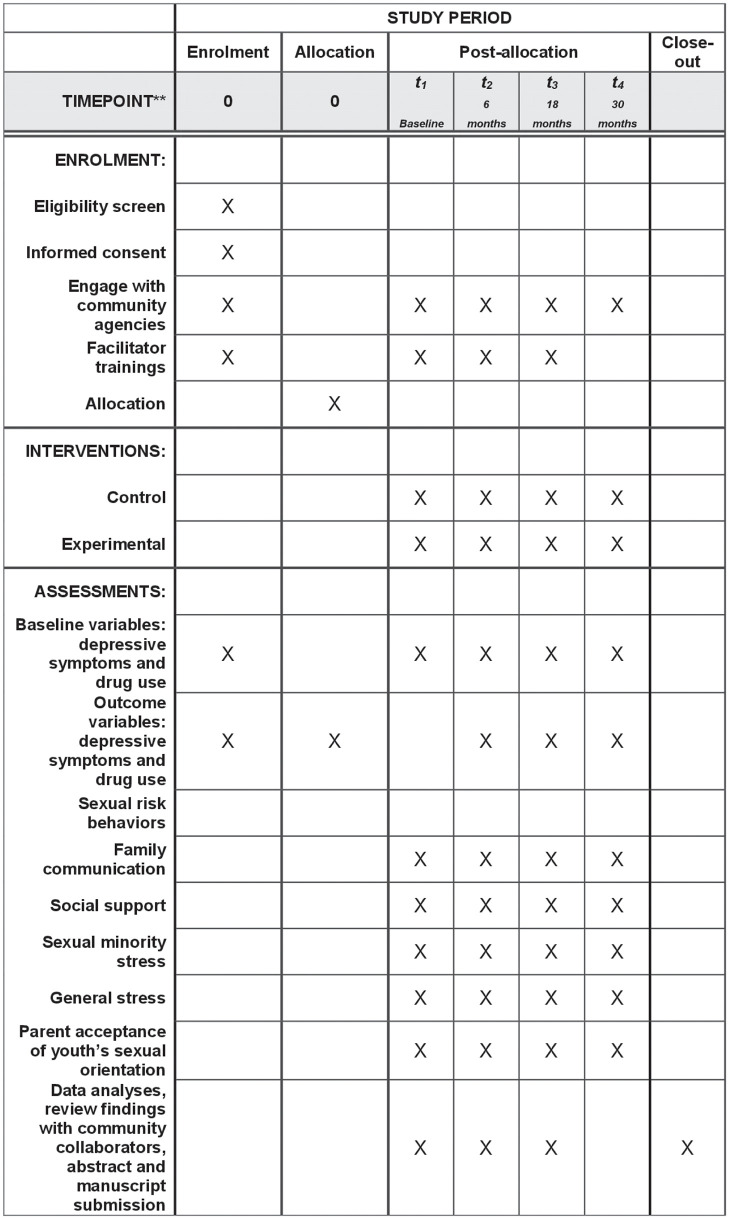
Schedule of enrollment, intervention, and assessments.

This study was approved on April 13, 2023, by the University of Miami Human Subjects Research Office, study # 20230372. The enrollment schedule, intervention, and assessments are available in Fig 1. During the time of manuscript preparation, participant recruitment had not yet begun. The authors confirm that all ongoing and related trials for this intervention are registered. Recruitment is scheduled to last 21 months from January 2024 and follow-up assessments will run through December 2027.

### Study design

This study uses a randomized trial design with two arms (Familias con Orgullo and community practice) as the between subjects factor with four assessments at baseline and 6-, 18-, and 30-months post baseline as the within subject factor. Dyads of Hispanic youth (*N* = 306) and their primary caregivers (i.e., parents, which may include: mother, father, or legal guardian) will be randomly assigned to Familias con Orgullo or the community practice condition. Participants will be randomized on a 1:1 randomization ratio. An urn randomization will ensure comparability across conditions on the following variables (see measures section below): (1) current drug use (yes/no) and (2) level of depressive symptoms (below 16 on the CES-D or greater than or equal to 16).

### Participants and inclusion criteria

The sample will consist of 306 Hispanic sexual minority youth and their primary caregiver who meet the inclusion/exclusion criteria, which will be verified by a brief screening measure that has been successfully pilot tested. Participating families must meet the following criteria: 1) Youth, 13–17 years of age, who report at least one of the following: a) identify as gay, lesbian, or bisexual, or b) reports same-sex sexual behavior, 2) Youth has disclosed their sexual minority status to at least one parent, 3) Youth is of Hispanic immigrant origin, defined by having at least one parent that self-identifies as Hispanic (English and Spanish speaking Hispanics can participate in the study), 4) Youth lives with an adult parent who is willing to participate, and 5) Family lives in South Florida. Families will be excluded if the Youth identifies as transgender and if the family plans to move out of South Florida during the study period. We decided to exclude transgender youth because from our qualitative research with HSMY, which included a small number of transgender youth, we received feedback that Familias con Orgullo did not have a focus on gender identity, it lacked important components, and did not meet the needs of transgender youth (despite the focus on effective communication and parental support). Transgender youth who may be screened will be provided referrals to community services tailored to transgender youth.

### Familias con Orgullo

Familias con Orgullo is a multi-level, family-based intervention that targets various risk and protective factors across the different contexts that impact youth and families. For further information on the intervention development process and detail on the intervention content, please see Lozano et al. [7]. Familias con Orgullo was iteratively developed based on feedback from HSMY and their parents [7] and targets the synergistic influence of drug use and depression through improvements in family functioning, parent support for the Youth, and parent acceptance. Unlike most interventions with SMY, Familias con Orgullo places parents in the role of change agent for the family. Specifically, the parent makes changes on behalf of the family and supports changes that youth make for themselves that subsequently impacts their outcomes [7]. Further, youth learn skills to manage sexual minority stressors through emotion regulation, role plays, and development of social support networks. Familias con Orgullo consists of 14 sessions (Table 1): seven multi parent group sessions, three multi youth group sessions, and four family sessions per parent-youth dyad.

**Table 1:** Familias con Orgullo—Session by session overview.

Session	Description
**Family Session #1—Engagement Family Session**	Engagement of both parent and youth by applying sexual minority affirmative counseling practices, and meeting families where they are regarding acceptance of sexual minority disclosure. Problem-solve family’s perceived barriers to participation.
**Parent Group Session #1—Parental Investment in Adolescent Worlds:**	Introduction to Familias con Orgullo and review of youth sexual minority stressors in family, school, and peer worlds. Parents are made aware that disclosure and/or acceptance is a process for both parents and youth. Parents learn about drug use and depression, its contributing factors and the role of family and parenting in countering these effects (e.g., support, acceptance).
**Parent Group Session #2—Enhancing Communication and Supportive Relationships**:	Focuses on the characteristics of effective family communication and parents engage in an exercise to reinforce key communication skills. Parents discuss what it means to be supportive and accepting of sexual minority youth.
**Adolescent Group Session #1—Effective Communication, Sexual Minority Stress, and Goals:**	Review of youth stressors in the family, school, and peer worlds, how to manage these stressors, and goal setting. Youth learn the characteristics of effective communication and role play the discussion of a difficult topic with parents.
**Family Session #2—Family Communication:**	Parents and youths use newly learned communication skills and practice by discussing a relevant issue in the youth’s life related to being a sexual minority.
**Parent Group Session #3—HSMY Empowerment:**	Highlight the role of supportive parents and accepting behaviors toward youth and how these impact HSMY health. Provides parents with emotion regulation skills and how to help youth regulate their emotional responses. Parents learn about depression in HSMY, the role of minority stressors on mental health, and how to be supportive in helping youth cope with depressive symptoms.
**Adolescent Group Session #2—HSMY Empowerment:**	Empowers youth with strategies to deal with emotional responses to chronic sexual minority stressors. Youth learn about the connection between thoughts, feelings, and behaviors and how they impact emotional health. Youth learn coping strategies such as identifying and modifying faulty thinking, breathing techniques, and problem solving.
**Parent Group Session #4—HSMY and Drug Use:**	Parents learn about drug use risks HSMY face and how sexual minority stressors (e.g., discrimination, homophobia) may contribute to drug use. Review with parents how drug use is a maladaptive coping strategy to manage sexual minority stressors. Highlights the role of pro and antisocial peers.
**Family Session #3—Parental Monitoring of Peer World and Drug Use:**	Parent talks with youth about the role of peers in promoting or discouraging HSMY drug use. Family discusses how youth can establish and maintain supportive and prosocial sexual minority peer networks and how parents can support these positive relationships.
**Parent Group Session #5—Adolescent Sexual Health: Dating and Dating Violence:**	Discussion of parental attitudes and beliefs about youth dating specific to sexual minority youth, such as bringing home a same sex partner and gendered Hispanic cultural beliefs that may impact parent-youth communication about sexual health. Parents learn about the unique and elevated risks faced by HSMY and positive ways that parents can influence youth behavior.
**Adolescent Group Session #3—Adolescent Sexual Health: Dating and Dating Violence:**	Youth explore and identify their attitudes and beliefs regarding dating and learn the effects of youth sexual risk behaviors. Discussion on components of a healthy romantic relationship and potential red flags for dating violence. Youth discuss sexual behavior expectations of sexual minority culture and how to manage pressure to engage in risk behaviors. Youth learn how they can promote safe relationships and safe sexual behaviors. Youth role play assertiveness skills around boundaries and expectations.
**Family Session #4—Adolescent Sexual Health:**	Parents communicate the dangers and consequences of risky sex. Parents help youth identify components of a healthy romantic relationship and parental expectations around sex. Parents guide youth in developing safety skills such as communicating with a partner about condom use and assertive limit setting.
**Parent Group Session #6—Parental Investment in Adolescent’s School and Adolescent Support Systems:**	Addresses the role of school in the youth’s life and how parental connections to school are a protective mechanism. Parents learn about school support systems (e.g., Gay/Straight Alliances) and community supports for SMY. Parents discuss ways to support youth when confronting discrimination from school staff and/or peers and ways that parents can be advocates for youth.
**Parent Group Session #7—Prevention Everyday:**	Highlights parents’ role as lifetime educators and advocates for their youth and the importance of daily implementation of skills. Parents review the interconnectedness among behaviors and the importance of communication, family support, and parental acceptance in combating risks.

The seven (2-hour) parent group sessions bring parents together to foster social support, family functioning, and increase parental support and acceptance for the youth. Through a participatory learning process, parents learn about conditions HSMY face and the impact on youth health. Facilitators build rapport and group cohesion to engender the group processes that will help facilitate parental support and acceptance of HSMY youth. Parent group sessions will raise parental awareness about not only the risks that HSMY face in today’s society, but also, the protective factors that are modifiable and well within parental reach to protect youth from risk and foster health. Behavioral rehearsal, such as role plays, and feedback are utilized to reach group goals and help parents develop skills for subsequent stages of the intervention. If there is more than one parent who wishes to participate in the Familias con Orgullo sessions, they can.

The three (2-hour) adolescent group sessions bring youth together for social support and for a conjoint skill learning process. The main goals of the adolescent group sessions are to build adolescent skills in confronting and managing stressors related to being a sexual minority. Youth learn skills such as emotion regulation, relaxation training, goal setting, and problem solving. Adolescents also learn how to protect themselves from drug use, in addition to how to foster their mental health. Further, the sharing of common experiences and group support are tools for fostering mental health as well. The facilitator and family [e.g., adolescent and parent(s)] participate in four (1-hour) family sessions, which create an opportunity for parents to transfer the competencies learned in the group sessions to their adolescent, foster supportive relationships, and increase parent-adolescent communication.

The control condition will consist of community practice with referral. Families randomized to this condition will be provided with information and referral regarding available services in the community that focus on supporting sexual minority youth and their families. Our team has compiled a list of available services such as mental health services, support groups, and advocacy groups and organizations.

### Familias con Orgullo facilitator training, supervision, and fidelity

Familias con Orgullo will be delivered by bilingual and bicultural facilitators who have a degree in psychology, social work, or related background, and at least five years of experience working with Hispanic families and sexual minorities. Training and supervision for study facilitators will be performed by a licensed social worker with extensive experience in training and supervision who is the lead clinical supervisor for the study. The training will be a total of 24 hours delivered in a 3-day workshop for new facilitators and a booster training of 8 hours for previously trained facilitators (i.e., during the formative work for Familias con Orgullo [7]). Training approaches will include review of the intervention manual, didactic presentations, videotape reviews, and role-plays. The facilitators will also review strategies to build alliance with the families and receive reinforcement in affirmative practices and sensitivity training when working with HSMY. Supervision will consist of weekly 1-hour group supervision meetings. In these meetings, the lead clinical supervisor and facilitators will discuss and problem solve unique intervention and/or fidelity concerns. Additionally, group supervision for the family sessions may include presentations, fidelity and case review, and session planning.

To monitor implementation fidelity, 100% of group sessions and 25% of randomly selected family sessions will be rated by an independent fidelity rater. Additionally, 25% of sessions rated by the independent rater will be rated by research personnel to obtain inter-rater reliability. Previously used observational adherence measures from our Familias con Orgullo pilot study will be used to conduct adherence ratings. Key facilitator process behaviors (e.g., active listening, participant engagement, positive alliances) are measured on a scale from 0 to 6 with higher scores indicating better delivery; content items are dichotomous and ratings are based on whether the facilitator delivered the content or not. In our prior studies, inter-rater reliability has been > .75. Fidelity ratings will also be discussed with the facilitator during group supervision.

### Recruitment procedures

Participants will be recruited from the Miami-Dade County Public School system (MDCPS), local South Florida organizations that serve sexual minorities, social events attended by sexual minorities, social media, electronic flyers posted on community partners’ websites, and through word of mouth. We have previously collaborated with the MDCPS and the community agencies we will be working with in this study [7, 17]. A list of clinics can be found at ClinicalTrials.gov.

To recruit participants, trained assessors will approach families (i.e., adolescent and parent/legal guardian) and ask them if they are interested in seeing if they qualify to participate in a study about youth well-being and family relationships. If the family is interested in learning more and determining if they qualify for the study, they will be moved to a private area. The assessor will then screen the parent and youth. To protect participants’ sexual minority status, our screening measure also includes questions on levels of physical activity, family communication, quality diet, and substance use. Further, we do not explicitly state that our study has an HSMY focus because qualifying for the study would then identify the status of someone who may not want their sexual minority status revealed. The screening measure is administered via iPads and after the potential participant completes it, the participant will receive a message stating, “you are eligible for this study” or “you are not eligible for this study” based on scores calculated by REDCap, a secure HIPAA compliant software for data collection [18]. If eligible, participants will be informed about the study, including that they will be randomized to potentially participate in adolescent group and family sessions. In our past work with HSMY, many of our participants were referred to the study via word of mouth. In the case where a participant calls or indicates interest in the study, a recruiter will coordinate a time to meet the participant to screen, consent, and assent. Once parent and youth are consented/assented following the screening, they will complete a short assessment battery on a tablet. Following the assessment battery, the youth will be asked to provide a sample of their urine and hair for drug and cortisol testing which will be processed by a qualified lab. The family will then be randomized to one of the two study conditions and compensated for their time. Families are assessed at baseline, 6-, 18-, and 30-months post-baseline. Parents are compensated $55, $60, $65, and $70 cash, for each respective assessment; youths receive $20, $25, $30, and $35 cash, for each respective assessment. To promote participant retention and complete follow-up we will collect participant address, email and telephone where participants can be contacted for follow-up. Participants will also complete a detailed tracking form that includes three secondary contacts (e.g., family, friends) if we are unable to make contact for follow-up assessments.

### Data collection and measures

Consent, assent and survey measures will be available in Spanish and English. All survey-related materials will be completed using REDCap software. All assessments will occur in person. Data from the assessments will be stored in secure university servers and kept separate from identifying information. Moreover, the assessment data will be labeled with a numerical ID that is only accessible to approved personnel and is in a password protected database. All study measures, with the exception of the youth’s drug screen urine and hair cortisol samples, are self-reported and include: youth drug use, youth depressive symptoms, youth sexual risk behaviors, parent and youth family functioning, youth perceived social support, youth perception of parent acceptance, youth sexual minority and general stress. This study has a data safety and monitoring plan (DSMP). The study will be audited by the Quality Assurance (QA) Unit within the research team’s department. This unit is responsible for verifying compliance with regulatory and participant safety requirements ensuring that all aspects of protocol implementation are ready to initiate study enrollment and ensures routine monitoring of all data collected in the protocol. Deidentified research data will be made publicly available when the study is completed and published.

The *Center for Epidemiologic Studies Depression Scale* [19, 20] will be used to asses youth reported depressive symptoms. The CES-D asks how often a person felt symptoms of depression in the past week. The *Monitoring the Future* [21] survey will be used to assess youth drug use based on report of past 90-day drug use. If a youth endorses past 90-day drug use, they will be asked about frequency of use. The *Simple Screening Instrument for Alcohol and Other Drugs* (SSI-AOD) will assess drug use, preoccupation and loss of control, adverse consequences, problem recognition, and tolerance and withdrawal. Moreover, urine toxicology screening at each timepoint will serve as a check that can also enhance the accuracy of self-reported drug use [22, 23]. Drug use urine toxicology screening kits will screen for recent cocaine, methamphetamine, marijuana, opiate, and benzodiazepine use.

A secondary outcome in this study is related to youth sexual risk behaviors. Youth sexual risk behaviors will be assessed with items from the *Sexual Behavior Instrument* [24] to ask youth about condom use during last intercourse as well as the number of times, they have had sex (oral, vaginal, or anal sex) in their lifetime and in the previous 90 days. Additionally, youth are questioned about the occurrence and frequency of high-risk situations during sexual intercourse (i.e., condomless sex, being under the influence of drugs and/or alcohol during sexual encounters).

For hypothesized study mediators, the *Parenting Practices* [25] and *Parent-Adolescent Communication* [26] scales will be used to assess parent and youth reported family functioning. *The Multidimensional Scale of Perceived Social Support* [27] will examine perceived social support from family and friends and the *Perceived Parental Reaction Scale* [28] will assess youth perceptions of parental reactions to sexual minority disclosure. We will also measure youths’ sexual minority stress using the *Sexual Minority Stress Inventory* [29] and the *Perceived Stress Scale* [30] to assess general stress. Additionally, we will also use hair cortisol collection & assays to assess youth stress. Hair cortisol offers a retrospective record of systemic cortisol exposure over weeks to months. This longitudinal perspective is advantageous for capturing cumulative stress exposure, thus offering richer insights into the relationship between chronic stress and various health outcomes. Furthermore, the non-invasive nature of hair sampling makes it a practical and ethical choice for diverse populations [31]. The most proximal ~3 cm of the hair strand will be used for analysis to reflect approximately the last three months of cortisol exposure.

### Data analysis plan

#### Data preparation and preliminary analyses

The study statistician will have access to the final trial dataset. Testing of distributional assumptions will include statistical tests for univariate and multivariate normality (tests of skew & kurtosis) as well as visual inspections of the empirical distributions of the data at each time point. Should deviations be deemed sufficient for concern, transformation of variables will be attempted where possible. Reliability estimates of internal consistency (Cronbach’s alpha) will be generated for all scale scores. If reliability estimates are found to be below .80, item total correlations and factor analyses will be employed to diagnose and correct psychometric problems [32].

#### Measurement modeling

A family functioning latent construct will be created consisting of two indicators (i.e., parental involvement, and communication) to create a measurement model based on a confirmatory factor analysis using SEM techniques [33, 34]. Given that only two indicators will be used to create the latent family functioning construct, we will constrain the factor loadings to be 1 for model identification [35]. In our previous studies, we have consistently found large correlations (r > .6) between these two indicators.

#### Main effects

All analyses will be conducted in M*plus* which utilized Full Information Maximum Likelihood to handle missing data [36, 37]. To examine the efficacy of Familias con Orgullo, compared to community practice, in decreasing past 90-day drug use (frequency and quantity) and depressive symptoms among HSMY, over 30-months, we will use latent growth modeling (LGM) [38]. Growth models can be expressed in terms that are identical to multilevel models, where the first level fits each response in time to an individual-level growth model and the second level represents how the slope of outcomes (drug use and depressive symptoms) varies by condition. The second level represents the condition effect on the slope of outcomes (drug use and depressive symptoms). As recommended by Raudenbush and Bryk, we will use 2-level analysis to determine whether the mean trajectories of outcomes for Familias con Orgullo and control differ significantly over time [39]. To account for the non-independence of outcomes, we will conduct multivariate growth modeling similar to that used in evaluating a previous trial [40]. If correlations have above moderate effects (.30 < *r*) [41], we will empirically examine the distinct contributions of the intervention on each separate outcome, using false discovery rate to account for multiple comparisons. Data analysis for this hypothesis and all others will be conducted using M*plus* (v 8.8) [42].

M*plus* Monte Carlo simulation with 10000 replications [43] was used to calculate the power for the main effects outcomes using a latent growth curve model framework with one covariate (i.e., condition assignment) and missing data. With 4 time points and assuming equal sample sizes across both conditions (i.e., 153 cases in each condition) and 20% attrition rate, which is consistent with the attrition in our past studies [12], we have 80% power (assuming a Type I error = .05) to detect a regression coefficient equal to 0.12 (d = 0.36) in the regression of the slope growth factor (for both drug use and depression) on intervention condition. This is considered a small effect size [41, 43].

To test for mediation, we will use the "product of coefficients" test described by MacKinnon, which is based on the distribution of the indirect effect of the intervention through a mediator [44]. This procedure tests whether the product of the coefficients from the intervention to the mediator (a) and from the mediator to the outcome (b) is significantly different from zero. Specifically, we will estimate paths between the intervention condition and mediators (a path), and between mediators and outcomes (b path). Because we have four time points, our first mediation analyses will fit bivariate growth curves for both mediators and outcomes and examine the role of the (latent) slope of mediators on the slope of outcomes. The product of the two pathways (path ‘a’)*(path ‘b’) is the indirect effect of Familias con Orgullo to each of the outcome trajectories through the mediators. As noted above, we will be testing whether the product (a)*(b) is statistically significantly different from zero, by comparing the observed value of (a)*(b) to the empirical distribution calculated using the bias-corrected bootstrap [45]. In addition to the mediation models, we will also test if the initial changes in mediators from baseline to the 6-month follow-up mediate the effect of intervention condition on each of the outcome trajectories.

For the mediation analysis, we used M*plus* Monte Carlo simulation with 10000 replications to calculate the power using a mediation model framework [46]. With a baseline sample size of 306 (assuming 20% attrition rate at each assessment time point), we have 80% power (assuming a Type I error = .05) to uncover a significant mediating effect when the regression coefficient of pathways ‘a’ is approximately 0.37 and ‘b’ is approximately 0.23 (i.e., R^2^ effect size were 0.27 for the relationship between intervention and mediator; i.e., path ‘a’) and mediator and outcome (i.e., path ‘b’), which is considered a small effect [41, 43].

To test whether gender and baseline levels of parent support for the youth, parent acceptance, family functioning, youth stress, and sexual minority stress moderate intervention effects on the outcomes, an interaction term between intervention condition and each of the potential moderators will be created and entered into a growth curve model by regressing the trajectory slope on condition, gender, and the potential baseline moderators, and their interaction. For probing the interaction effect, the Johnson-Neyman (JN) technique [47] will be used to assess where moderation occurred. The JN technique identifies regions of significance of intervention effects on the outcome trajectory. Based on the results of the JN technique, we will estimate two separate trajectory models to examine the intervention effects on each of the outcome trajectories. Also using Monte Carlo simulation analyses with 10000 replications, power analyses were conducted for this hypothesis using a moderation model framework. With a baseline sample size of 306 (assuming 20% attrition rate at each assessment time point), we have 80% power to uncover a significant moderation effect (p < .05) when the regression coefficient of the interaction is approximately |0.14| (i.e., *R*^*2*^ effect size were |0.02| for interaction effect), which is considered a small effect [41].

In terms of post-hoc analyses, because we will measure past 90-day frequency and quantity of alcohol and cigarette use, we will examine condition effects on these outcomes. We will also examine intervention effects on sexual risk behaviors (i.e., condomless sex, number of sexual partners, and sex while under the influence of drugs and/or alcohol). As such, we will analyze youth differences in outcomes by these subgroups as well as differences in the mediation pathways. Using the same LGM approach described, we will also explore intervention effects on secondary outcomes of past 90-day frequency and quantity of alcohol use and cigarette use as well as condomless sex (as applicable), number of sexual partners, and sex while under the influence of drugs and/or alcohol. Across all analyses, to account biased estimation of intervention effects influenced by low base rates, we will adjust standard errors of intervention effects on the slope of outcomes by using a Huber-White ‘sandwich’ estimator [48]. We will also explore differences among subgroups of HSMY who may be at higher risk for drug use and depression symptoms: US born, acculturated Hispanic youth, and low SES families.

## Discussion

In this manuscript, we described the rationale, methods, and design of a randomized controlled trial testing the efficacy of Familias con Orgullo, a family-based intervention designed specifically for HSMY and their families for the prevention/reduction of drug use and depressive symptoms among HSMY. Such interventions that are tailored to the unique needs of HSMY are necessary given the saliency of family and Hispanic cultural values and beliefs for HSMY. The efficacy study described begins to fill a critical gap in the intervention literature because few existing interventions for sexual minority populations have been rigorously evaluated in randomized controlled trials; Familias con Orgullo will be the first family intervention evaluated with HSMY and their families. Familias con Orgullo utilizes parents as the agents of change in preventing and decreasing drug use and depressive symptoms among HSMY. This is notable because current interventions are mainly individual level, and while a few include parents as the agent of change for their youth in the intervention process, none do so with HSMY. Moreover, the interplay of cultural values, minority stressors, family dynamics, and inter- and intra- personal level variables that impact HSMY health will be accounted for and targeted in Familias con Orgullo. Importantly, this intervention was user-centered, such that it was developed based on feedback from HSMY and their parents, ensuring that it is applicable and relevant to their needs.

This study, if successful, will provide evidence that Familias con Orgullo can effectively prevent/reduce drug use and depressive symptoms among Hispanic sexual minority youth. The importance of evaluating interventions such as Familia con Orgullo is highlighted by the National Academies of Sciences, Engineering, and Medicine’s call for research that examines family relationships to further enhance understanding of the well-being of sexual minority populations [49]. Establishing the efficacy of Familias con Orgullo will also pave the way for Familias con Orgullo to be further evaluated, and ultimately implemented and disseminated into community practice, so that it is the standard of care for Hispanic sexual minority youth and begins to ameliorate health disparities related to drug use and depression that impact this vulnerable population.

## Supporting information

S1 Checklist Spirit 2013 checklist. (DOC)

S1 File Study protocol. (PDF)

S2 File Parent consent. (PDF)

S3 File Adolescent assent. (PDF)

S4 File Biological specimens. (DOCX)
