# Supplementary material for: Familias con Orgullo: Study protocol for an efficacy study of a family-based intervention for Hispanic sexual minority youth
Source: PLoS One. 2023 Dec 15;18(12):e0295683. doi: 10.1371/journal.pone.0295683 (PMC10723681; doi:10.1371/journal.pone.0295683)
Supplement: S1 File — (PDF) [file pone.0295683.s002.pdf]

Promoting Health and Reducing Risk among Hispanic Sexual Minority Youth and their Families

PI: Guillermo "Willy" Prado, PhD

IRB #: 20230372

October 6, 2023

NCT #: NCT06057337

Version 3

1) Protocol Title

Promoting Health and Reducing Risk among Hispanic Sexual Minority Youth and their Families

2) Objectives*

This study will evaluate the effects of a parenting intervention for Hispanic sexual minority youth in preventing/reducing drug use and depressive symptoms. It will also examine whether the intervention improves parent social support for the adolescent, parent acceptance, family functioning, and whether it reduces general stress and stress associated with being a Hispanic sexual minority. The knowledge expected to be gained from this study has the potential to transform the type of prevention programs that are disseminated to communities to improve the health of Hispanic sexual minority youth.

AIM 1: Examine the efficacy of Familias con Orgullo (FcO), compared to community practice, in decreasing past 90-day drug use (frequency and quantity) and depressive symptoms among HSMY, over 30-months.

> **H1**: FcO will be efficacious, compared to community practice, in decreasing past 90-day drug use frequency and quantity and depressive symptoms among HSMY, over 30-months.

AIM 2: Examine whether the relationship between intervention condition and the outcomes are partially mediated by parent support for the adolescent, parent acceptance, family functioning, adolescent stress, and sexual minority stress.

> **H2**: The intervention effects on youth outcomes will be partially mediated by changes in parent support for the adolescent, parent acceptance, family functioning, adolescent stress, and sexual minority stress.

Exploratory AIM 3: Explore whether gender and baseline levels of parent support for the adolescent, parent acceptance, family functioning, adolescent stress, and sexual minority stress moderate intervention effects on the youth outcomes.

H3: Baseline levels of parent support for the adolescent, parent acceptance, family functioning, adolescent resiliency, adolescent stress, and sexual minority stress will moderate intervention effects on youth outcomes such that youth at higher risk (e.g., families reporting higher stress) will benefit most from FcO.[11,17] Consistent with our prior work, we do not expect differences by gender.

3) Background*

Sexual minority youth (SMY) in general, and Hispanic sexual minority youth in particular (HSMY), report higher levels of drug use and depressive symptoms, compared to their heterosexual and sexual minority peers.[1,3,18-23] For this study, SMY refers to adolescents who identify as a sexual minority (e.g., gay) or engage in sexual behavior with individuals of the same sex.[24] Given that there are approximately 3.2 million SMY in the US between 8 and 18 years old, HSMY represent approximately 21% of the US

total SMY population, and that 60-80% of youth have disclosed to at least one parent,[25] addressing these disparities is a public health priority that merits significant attention.

Despite a need, the majority of psychological intervention research does not generally include SMY, and most recommended adaptations are not empirically supported by rigorous research.[26] A review of the sexual minority youth intervention literature identified limited available interventions, the majority of which were individual level and excluded the family, a central component for HSMY, as part of the intervention process. Further, very little research incorporates the influence of family on the health of SMY;[27] the few that have, examined these variables cross-sectionally[28] and only from the youth perspective.[27] There are currently no family-based interventions for SMY in general,[27] or for HSMY in particular, and their families to prevent drug use and depressive symptoms. This is unfortunate given a rigorous body of research on the effects of family-based prevention programs in improving health outcomes for youth, including behavior problems, drug use,[9] and depressive symptoms;[17] and improving family relationships.[9,11,29] Interventions such as the Family Check-Up,[30] Familias Unidas,[9,13] and the Strong African American Families Program[31] improve health outcomes among youth, with long-term effects.[32]

The lack of scientific inquiry with HSMY is disconcerting because the limited available research shows that negative health outcomes are disproportionate and pervasive among HSMY. For example, almost twice as many HSMY (20.4%) reported drug use in the last 90 days compared to their non-Hispanic sexual minority counterparts (11.5%). Additionally, a recent study found that HSMY had higher odds (1.71 times) of attempting suicide compared to their non-Hispanic White counterparts.[18] Given these statistics, Hispanic adolescent sexual minorities are likely to evince these health disparities as well. The multiple adverse health outcomes affecting HSMY require intervention at multiple levels to address the complexity of experiences confronting this high-risk population. The interplay of racism, homophobia, cultural values, family dynamics, and intra- and inter- personal level variables are important considerations in interventions to decrease health disparities and negative health outcomes among HSMY. For example, it is important to address the health impacts of sexual discrimination and internalized homophobia on family relationships. These inter-related health outcomes, which often have common underlying factors, are a syndemic that act synergistically[33,34] to disproportionately impact youth.

Syndemics theory, the study of co-occurring epidemics and their epidemiological relationships on disease outcomes in marginalized populations,[35,36] can help delineate the root causes of health disparities and negative health outcomes in HSMY. A syndemics model highlights that health outcomes (e.g., drug use and depression) are not distinct from each other but interact synergistically. Further, sexual minority stressors such as discrimination and internalized homophobia have a critical effect on the development of syndemics among stigmatized groups[37] such as HSMY. Hispanic sexual minority youth face the simultaneous stressors of racism and homophobia, which compound the effects of behavioral health disparities.[38] The additive impact of syndemic indicators is associated with adverse health outcomes.[38,39] Under this framework, the relationship between marginalized populations such as HSMY and the numerous challenges they

endure as a result of being exposed to multiple sexual minority stressors (e.g., discrimination) and interpersonal stressors (e.g., lack of parental acceptance) can impact family functioning and exacerbate health disparities.[40,41] It is necessary to understand contributors to these syndemic outcomes from multiple levels, while accounting for the unique experiences of HSMY and their families, including the interplay of cultural values, vis a vis family-based intervention. It is critical to develop and test preventive interventions that will mitigate the unique risk factors contributing to the negative health outcomes disproportionately experienced by HSMY[42,43] to increase well-being among HSMY and their families.

Family functioning has a central role in countering or amplifying the disparities faced by HSMY. While families may be a source of support for HSMY, they may also be an additional source of discrimination and rejection.[44] Family level dynamics such as parental support and parental acceptance play a crucial role in the health of HSMY through the disclosure process and beyond. Although parental and family support predict the general well-being of SMY, even in the absence of other types of support,[45,46] HSMY report higher rates of negative reactions[3] and lack of support,[47] compared to non-Hispanic Whites, upon disclosing to their families. Lack of parental support in disclosing to parents has been associated with increased odds of depression and drug use in population based surveys.[48,49] Alternatively, family support among HSMY has been associated with higher self-esteem,[50] less depressive symptoms, and fewer suicide attempts.[5] Further, research has found that perceived family support is the most powerful predictor in HSMYs' decision to disclose to people in their lives.[51] Non-disclosure to parents may be associated with elevated levels of drug use and depression.[49]

Similarly, parental acceptance, or lack thereof, also impacts HSMY wellbeing. Parental acceptance has a protective effect on mitigating the risks HSMY confront,[52-54] whereas a lack of parental acceptance has been positively related to depressive symptoms.[55] Families can be a source of taunting, bullying, and commentary about mannerisms.[56] Further, stigmatizing verbal messages from family members, particularly name calling and labeling, are associated with low self-esteem and elevated shame.[57] The struggle to cope with these family dynamics can lead to depression[58] and drug use.[59] Taken together, lack of acceptance from Hispanic parents may partly explain why health risk behaviors are higher for HSMY who disclose to their parents.[3,55] Further, given the saliency of family among HSMY, it is important to intervene at the family level and to empower parents to be change agents that positively alter health outcomes for HSMY.

Hispanic parents in particular tend to have intolerant attitudes towards sexual minorities[60] and coming to terms with youth being a sexual minority can potentially lead to decreased family functioning. Parents may also be unaware of how to be supportive and accepting and may not know how common Hispanic cultural beliefs may impact the family's acceptance and support of SMY. For example, adherence to traditional gender roles such as machismo and marianismo,[61] have consistently been correlated with detrimental attitudes towards sexual minority groups.[62,63] Additionally, internalized homophobia may be associated with Hispanic cultural values around what it means to be a man. It is plausible that parental rejection in Hispanics may be due to Hispanic parents'

overall negative attitudes towards homosexuality,[47] religious beliefs,[64,65] cultural values, and expected gender roles ubiquitously present in Hispanic culture.[66] Perhaps parents may have difficulty accepting HSMY's disclosure because they grieve the idea of who their adolescent once was or because they worry about the new social challenges an adolescent may face after disclosure.[42] In addition to a possible shift in the parent-adolescent relationship, parents may also have to cope with changes in their relationships with others, such as with a co-parent, their extended family and their religious community.[42] Given the influence of Hispanic cultural values, it is therefore no surprise that some Hispanic parents have a difficult time with having a sexual minority youth, which consequently leads to elevated health risk outcomes.[3,55] Hence, among HSMY who have disclosed, facilitating the disclosure process and its aftermath is critical for stymying adverse health outcomes.

Considering that health disparities for HSMY are partly attributed to parental lack of support and acceptance after disclosure, family-based interventions may provide crucial support to HSMY and their parents. Family-based interventions are among the most efficacious and effective interventions in the prevention and reduction of drug use and adverse mental health outcomes for adolescents in general.[9,11,12,67-69] Extensive research has established the crucial role that Hispanic parents play in healthy adolescent development and the prevention of health risk behaviors,[11,17] including drug use[9] and psychological difficulties.[70] Additionally, due to its proximal nature, the family is also a strong influence in an adolescent's course of development.[71] However, despite the effectiveness of existing evidence-based family-centered interventions for youth in general, family based-interventions for HSMY who have disclosed to at least one parent have not been developed or evaluated. Family functioning constructs such as parent-child communication, which have provided the greatest impact on youth outcomes in family-based interventions,[72] need to be evaluated and tailored to the needs of parents of HSMY.

Preliminary Findings. We have substantial experience in developing and fielding family-based prevention trials for Hispanic families. Over the past 20 years, we have recruited over 6,600 families including families with sexual minorities. We have retained 80% of participants throughout 24-months or longer. We have been productive in disseminating results from these trials, producing more than 325 peer-reviewed articles from past field trials in the past 16 years in journals such as *JAMA Pediatrics*. These published studies provide strong evidence for our interventions with both Hispanic families and sexual minorities with outcomes such as drug use and depressive symptoms, both of which are targets of the proposed application. Below we describe findings that highlight our intervention research with Hispanic families, relevant studies with sexual minority youth, and the qualitative and quantitative findings that led directly to this application.

<u>Intervention Research with Hispanic Adolescents and their Families</u>. Prado and colleagues evaluated the efficacy and effectiveness of Familias Unidas (FamU), a Hispanic specific, family-based intervention, in three randomized clinical trials.[9,11,12] In these three published trials with Hispanic youth (ages 13 – 17), FamU was efficacious/effective in preventing and reducing drug use, and sexual risk behaviors. Moreover, although FamU did not directly address internalizing symptoms and

depression, it had crossover effects on both internalizing symptoms[78] and suicide ideation.[79] FamU effects have been partially explained by improvements in family protective factors (e.g., parent-adolescent communication). This rigorous body of research has established that FamU, which was highlighted in the National Academies of Science, Engineering, and Medicine 2019 prevention report,[80] is efficacious and effective in the reduction and/or prevention of adolescent health problems. Importantly, FamU has moved from research to practice and is currently being widely disseminated in multiple states in the U.S. (e.g., RI, PA, and NY) and in Chile.

Descriptive Studies of Hispanic Sexual Minority Youth. Ocasio, Prado, and colleagues examined substance use and risky sexual behavior rates in HSMY (n = 194) living in Miami, FL compared to their heterosexual (n = 1,437) counterparts.[1] HSMY were identified as self-reported anal/vaginal/oral sex with a partner of the same sex. HSMY endorsed significantly more substance use than non-HSMY, including lifetime cigarette (32.1% vs. 13.1%; $p < .001$) and illicit drug use (26.4% vs. 11.4%; $p < .001$). Adjusted odds of lifetime use for all substances and past 90-day cigarette use (aOR = 3.07; 95% confidence interval: 1.50–6.31) were also significantly higher in HSMY, compared to non-HSMY. In a separate study, Ocasio, Prado, and colleagues showed that HSMY also reported significantly higher depressive symptoms than non-HSMY for those exhibiting clinical level symptoms of depression (aOR = 2.29; 95% CI: 1.18-4.47).[75]

Efficacy Study of FamU with HSMY Subsample. Ocasio, Prado, and colleagues assessed the relative efficacy of FamU in improving drug use and condomless sex in a sample of 194 HSMY.[15] Participants were randomized to FamU (n = 94) or treatment as usual (n = 100). The results showed that FamU was efficacious, relative to control, in improving parent-adolescent communication (β = 0.51; [0.41, 0.62]). There were no significant effects on drug use which speaks to the importance of developing interventions, such as Familias con Orgullo, specifically for Hispanic sexual minority youth.

Formative Work Including Feasibility and Acceptability of FcO. Development of Familias con Orgullo (FcO) was participant-focused, iterative, and multi-staged. The development process consisted of two phases: (1) formative work via individual interviews with parents and adolescents to inform initial intervention content,[8,16] and (2) feasibility and acceptability pilot of FcO.[7] First, qualitative individual interviews with 15 HSMY adolescent and parent days were conducted to inform FcO.[16] The individual interviews aimed to clarify the disclosure process for HSMY and their parents and how the disclosure affected parent-youth relationships and communication.[16] Participants were recruited from community agencies that serve sexual minority youth, through word of mouth, and social events for sexual minority youth. Interviews lasted approximately 60 minutes and were audio recorded and transcribed verbatim. Three themes were derived from the data: (1) intrapersonal challenges, (2) navigating disclosure, and (3) conceptualizing acceptance. Additional individual interviews with 12 adolescent parent dyads were conducted to elicit feedback on specific program components for a family-based intervention for this population.[8] Findings from the interviews suggested that interventions for HSMY should address the barriers to disclosure, such as Hispanic cultural values, to facilitate communication around the adolescent being a sexual minority. Participants stated that intervention content should be delivered face-to-face and that topics such as communication, emotional regulation, and sexual health should be

delivered to parent-only and adolescent-only groups separately before being addressed in family sessions. Participants also indicated the need for family sessions to discuss topics specific to individual family needs such as bringing someone of the same gender to family events. Further, some participants felt that participation in a family-based program for HSMY may depend on parental stage of acceptance. Based on these findings, we developed FcO, a family-based preventive intervention for HSMY and their families to prevent drug use and depression in HSMY.

To evaluate the feasibility and acceptability of FcO in a sample of 30 HSMY (a distinct sample from the HSMY and parents participating in the interviews described above) across three cohorts (mean age = 15.53, *SD* = 1.15) and their primary caregivers (from this point forward, parent) we conducted a pilot study using a randomized clinical trial.[7] Families were randomized to FcO or a no intervention control condition (i.e., community practice, including participant referrals) after parents and youth completed the baseline assessment. We aimed to: (1) assess the feasibility of recruiting HSMY and their parents, (2) assess the acceptability of the intervention from both parent and adolescent perspectives, and (3) pilot test the intervention and study procedures. An iterative, user-centered approach was utilized to enhance and modify FcO after the intervention was delivered to the first two cohorts based on feedback and suggestions about how to improve the intervention.[8] Following the first cohort of participants (n = 12; FcO n = 8, control group n = 4), we found that parents and youth were both enthusiastic and appreciative of the intervention, but youth requested an additional session specifically related to dating among sexual minority. Given this information, we developed an additional adolescent group session related to dating and sexual health. This new session was incorporated in the second cohort (n = 8; FcO n = 6, control n = 2) and we received positive feedback on that session, and the entire intervention, from both parents and youth. No additional content changes were made after the delivery of FcO with the second cohort of participants. Thus, participants in the third cohort (n = 10; experimental group n = 5, control group n = 5) received the same intervention as those in cohort 2. No changes were made after cohort 3.

To recruit study participants for each of the three cohorts, our study team interfaced with local community agencies that serve sexual minority youth. Agency staff let the study team know if there were eligible HSMY utilizing their services. Parent-youth dyads were eligible to participate if: (1) the youth had disclosed to at least one parent, (2) the youth was between the ages of 13 – 17, (3) the parent was of Latin American origin, (4) the youth reported same-sex behavior (or behavior with both sexes) and/or identified as gay, lesbian, or bisexual. A total of 30 families (of the 42 approached) met the inclusion/exclusion criteria and were enrolled. The high proportion (71%) of approached families who agreed to participate in this pilot highlights the feasibility of recruiting HSMY and their families into the proposed trial. After families were deemed eligible, they signed informed consent (parents) and assent (youth). Parents and youth then completed the baseline assessment and were compensated $60 (parents) and $30 (youth) for their time; participants also completed post-intervention assessments (at 6-months post baseline). Measures collected from the youth included: family functioning, drug use, depressive symptoms, sexual minority stress, condom use, and suicidal ideation. Parent measures included demographics and acculturation. Participants were not compensated

for attending intervention sessions. Additionally, we were able to successfully assess 100% (i.e., 30 of 30) of families through the post-intervention assessment.

At baseline, youth were, on average, 15.53 years of age (*SD* = 1.15). Additionally, 37% reported lifetime illicit drug use, 47% reported lifetime suicide ideation, 10% reported a suicide attempt in the past year, and 12% reported having had sex at least once in their lifetime. Finally, 50% of HSMY participants scored 16 or greater on the Center for Epidemiological Studies – Depression (CES-D) which is the cutoff used to identify individuals at risk for clinical depression. Findings[7] showed promising effects favoring FcO on parent adolescent communication (*d* = .46) and parental involvement (*d* = .34). There were also promising effects favoring FcO on suicidal thoughts [OR = .75] and depression symptoms [OR = .69]. Finally, all the adolescents in FcO either continued to remain drug free or transitioned from current use to no use (from baseline to post-intervention) compared to 74% in the control. There were no differences in effects on condomless sex [OR = 1.03] which is the reason we elected (per the reviewers' recommendation) to make this a secondary outcome instead of a primary one. Importantly the lack of findings in this outcome may be explained by two related reasons. First, only 12% of the sample reported engaging in sexual behavior at both baseline and 6-months post intervention. Second, our family-based intervention studies, and those of others, typically have delayed intervention effects on condom use and other sexual risk outcomes.[9,12,14,81,82] In fact, in both our efficacy and effectiveness studies of FamU, effects on sexual risk behaviors (including condom use) did not emerge until 30-months post baseline.[9,12,14] Even though only three sessions in FcO focus on sexual health (i.e., one parent group session, one adolescent session, one family session), based on our previous research, we expect that we will have an impact on sexual risk outcomes, therefore the intervention was not modified further. FamU only had two sessions specifically focused on sexual health and yet we observed intervention effects on sexual risk outcomes. Moreover, the effects were partially mediated by improvements in family functioning (e.g., parent-adolescent communication).[9,12] Nonetheless, sexual risk behaviors outcomes are not primary outcomes but will be collected and analyzed as secondary outcomes.

4) Inclusion and Exclusion Criteria*

Inclusion criteria:

1) Youth, 13 – 17, who report at least one of the following: a) identify as gay, lesbian, or bisexual, or b) reports same-sex sexual behavior;
2) Adolescent has their disclosed their sexual minority status to at least one parent;
3) Adolescent is of Hispanic immigrant origin, defined by having at least one parent that self-identifies as Hispanic (English and Spanish speaking Hispanics can participate in the study);
4) Adolescent lives with an adult parent who is willing to participate; and
5) Family lives in South Florida.

Exclusion criteria:

1) Adolescent identifies as transgender, and
2) Family plans to move out of South Florida during the study period.

5) **Procedures Involved***

Design. To evaluate the efficacy of Familias con Orgullo (FcO) in preventing drug use and depressive symptoms, this study will use a randomized trial design with two arms (FcO and community practice) as the between subjects factor with four repeated measures assessments at baseline and 6-, 18-, and 30-months post baseline as the within subject factor. In our prior studies we see changes in our proposed family functioning mediators at 3-months post intervention which would align with the 6-month follow-up. Additionally, we opted for an extended 30-month post baseline assessment period to allow for potential delayed effects of the intervention, as previously seen in our and other family-based interventions.[12,14,79,82] The 30-month follow-up was selected because this is the minimal time frame where we have observed effects on drug use.[12,14] Finally, an 18-month post baseline assessment was selected to avoid a 24-month gap between the 6- and 30-month follow-up assessments.

Participants. We will randomize 306 HSMY and their parents who meet the inclusion/exclusion criteria, which is verified by a brief screening measure that has been successfully pilot tested. An urn randomization will ensure comparability across conditions on the following variables: (1) current drug use (yes/no) and (2) level of depressive symptoms (below 16 on the CES-D or greater than or equal to 16). Randomization will be conducted by Dr. Brown, the biostatistician, after participant completion of the baseline assessment. A 1:1 randomization ratio will be used. Assessors will be blinded to randomization assignment.

Research personnel will wait to be approached and/or contacted by adolescents who have been given the study flyer. Interested participants will be invited to be screened for a study about adolescent well-being and family relationships. If the parent/adolescent is interested in participating, we will request verbal consent/assent. The assessor will explain the study. We are requesting permission to only utilize a verbal consent/assent for the screening measure portion, since it will be used to verify the inclusion/exclusion criteria. To protect participants' sexual minority status, our screening measure also includes questions on levels of physical activity, family communication, quality diet, sexual orientation, and drug use. These questions were included to compare adolescents who decide to participate in the study versus those who decline participation. Additionally, a variety of questions will help to minimize being singled out as a potential sexual minority. The screening measure is administered via iPads and after the potential participant completes it, the participant will receive a message stating, "you are eligible for this study" or "you are not eligible for this study."

Intervention Conditions

Familias con Orgullo (FcO).

All group and family sessions will be recorded. Participants who wish to not be recorded can move outside the range of the video camera. Recordings are necessary to detrermine facilitator behaviors and measure fidelity.
FcO aims to prevent the co-occurring epidemics of drug use and depression in HSMY by improving parent support for the adolescent, parent acceptance of the adolescent, family functioning (e.g., communication), and decreasing stress and sexual minority stress. Given the multiple health outcomes that HSMY face, and the multiple levels at which sexual minority stressors operate, the FcO intervention uses syndemics theory as a framework to conceptualize youth health. To account for the various levels at which challenges for HSMY occur, FcO is based on ecodevelopmental theory[84] and incorporates minority stress theory[85] to conceptualize youth outcomes. Beginning with the macrosystem, FcO addresses the broad societal factors and philosophical ideals that define a particular culture and impact adolescent health, such as Hispanic cultural values and norms that influence parent support and acceptance, sexual discrimination, and homophobia. Next, the intervention focuses on the microsystem in which the adolescent participates directly and impacts family functioning, family support, and acceptance. Finally, through the adolescent and family sessions, the intervention targets adolescent intra-individual level factors such as cognitive, affective, and behavioral responses, including internalized homophobia, which directly influence adolescent sexual minority stressors. The intervention targets drug use and depression through establishment of supportive relationships, managing minority stressors and their influence on health, and fostering positive family functioning which are all related to drug use and depression. These theories help frame prevention intervention efforts by addressing the multiple risk and protective factors operating at multiple ecological levels of sexual minority adolescents' intrapersonal and interpersonal contexts that influence drug use and depression.

FcO is a family-centered, multi-level intervention that targets risk and protective factors across multiple systems that affect youth and families. FcO targets the synergistic influence of drug use and depression through improvements in family functioning. Unlike most interventions with SMY, FcO places parents in the role of change agent for the family. Specifically, this means that youth outcomes are targeted through changes that the parent makes on behalf of the family, and through supporting changes that youth make for themselves. For example, the parent learns how to become a support and an advocate for the youth through affirmative practices and helps youth be a self-advocate. Further, youth learn skills to manage sexual minority stressors through emotion regulation, role plays, and development of social support networks. FcO consists of 14 sessions: seven multi parent group sessions, three multi adolescent group sessions, and four family sessions per parent-youth dyad. Two facilitators deliver each of the parent and adolescent group sessions to 12 to 15 participants per group. Each facilitator is responsible for conducting the four family sessions with half of the families in each group.

Parent Group Sessions. The seven (2-hour) parent group sessions bring parents together to foster social support, family functioning, and increase parental support and acceptance for the adolescent. Through a participatory learning process, parents learn about conditions HSMY face and the impact on youth's health. Facilitators build rapport and

group cohesion to engender the group processes that will help facilitate parental support and acceptance of HSMY youth. Parent group sessions will raise parental awareness about not only the risks that HSMY face in today's society, but also, the protective factors that are modifiable and well within parental reach to protect adolescents from risk and foster health. Behavioral rehearsal, such as role plays, and feedback are utilized to reach group goals and help parents develop skills for subsequent stages of the intervention. If there is more than one parent who wishes to participate in the FcO sessions, they can.

Adolescent HSMY Group Sessions. The three (2-hour) adolescent group sessions bring youth together for social support and for a conjoint skill learning process. The main goals of the adolescent group sessions are to build adolescent skills in confronting and managing stressors related to being a sexual minority. Youth learn skills such as emotion regulation, relaxation training, goal setting, and problem solving. Adolescents also learn how to protect themselves from drug use, in addition to how to foster their mental health. Further, the sharing of common experiences and group support are tools for fostering mental health as well.

Family Sessions. The facilitator and family [e.g., adolescent and parent(s)] participate in four (1-hour) family sessions, which create an opportunity for parents to transfer the competencies learned in the group sessions to their adolescent, foster supportive relationships, and increase parent-adolescent communication.

Control Condition. The control condition will consist of community practice with referral. Families randomized to this condition will be provided with information and referral regarding available services in the community that focus on supporting sexual minority youth and their families. Our team has compiled a list of available services such as mental health services (e.g., YES Institute), support groups (e.g., PFLAG), and advocacy groups and organizations (e.g., the alliance for GLBTQ Youth).

Measures.

Adolescent Measures

**Measure**
**ADOLESCENT**
**Primary Outcomes**
CES-D
Past 90-day Substance Use
Simple Screening Instrument for Alcohol and Other Drugs
**Secondary Outcomes**
Sexual Risk Behaviors
Suicide
**Protective/Risk Factors**
Parental Involvement
Parent-adolescent Communication

Multidimensional Scale of Perceived Social Support
Perceived Parental Reactions Scale (PPRS)
Sexual Minority Stress Inventory
Perceived Stress Scale
Vancouver Index of Acculturation
ACES
Bullying
Opioid Use
Youth Self Report (Externalizing Only)
CASA
European Cyberbullying Intervention Project Questionnaire
Expectations of Discrimination
Attitudinal Familism Scale
The Santa Clara Strength of Religious Faith Questionnaire
Sexual Orientation Discrimination
LGBT Social Justice Scale
SGM ACEs Scale
**Intra-Individual Factors**
Coming Out Timeline
Coming Out Growth Scale (COGS)
Emotional Expression Scale (EESC; Emotional Regulation)
The Brief Resilience Scale (BRS)
**PARENT**
Perceived Parental Reactions Scale (PPRS)
CESD
Parental Involvement
Parent-adolescent Communication
Vancouver Index of Acculturation
Revised Problem Checklist (Conduct Problems Only)
ACES
CASA
Parental Stress and Coping Inventory (PSCI)
The Santa Clara Strength of Religious Faith Questionnaire
Multidimensional Scale of Perceived Social Support<br>Attitudinal Familism Scale<br>LGBT Social Justice Scale<br>Latinx Caregiver Acceptance Scale

6) Data and Specimen Banking*

Hair Collection. Our team will implement established protocols for collecting hair samples which have been used in diverse populations around the globe with regards to age, gender, and sexual orientation. Below we describe the hair cortisol analysis:

1.1. Sample Collection and Storage

1.1.1. Label the outside of each tube and a plastic container with the following:
- 1.1.1.1. Individual/patient name or identifying ID number.
- 1.1.1.2. Date of Birth
- 1.1.1.3. Date of Collection
- 1.1.1.4. Time of Collection
- 1.1.1.5. Initials of Collector

1.1.2. Secure the entire length of hair to be sampled with a rubber band or clip.

1.1.3. Cut hair as close to the scalp as possible (taking care not to nick the skin) with clean scissors.

1.1.4. Place the hair sample in a pouch made of aluminum foil or a 15 ml screw-top polypropylene centrifuge tube.
- 1.1.4.1. Note: Samples can be stored indefinitely at -20 C and, if necessary, shipped overnight at ambient temperature.
- 1.1.4.2. Hair collection procedures should be practiced on volunteers, and the practice samples weighed. Hair samples as small as 5-10 mg can be analyzed; however, collecting samples > 10 mg is desired to minimize the likelihood of obtaining readings below the lowest CORT standard.

1.2. Transport to Biobehavioral Lab

1.2.1. Place the labeled bag in the refrigerator in the anteroom of room 340.
1.2.2. Record sample information on the log on top of the refrigerator.
1.2.3. Biobehavioral lab staff will retrieve samples and store them.

1.3. Sample Washing and Drying

1.3.1. Cut the hair sample into small pieces (1-3 mm)
1.3.2. Weigh the 2 ml Eppendorf tube.
1.3.3. Place into a 2 ml Eppendorf tube that is reinforced for bead beating.
1.3.4. Reweigh the tube to obtain the hair weight.
1.3.5. Record hair weight.
1.3.6. Add 1.0 ml isopropanol to each tube.
1.3.7. Place on the rotator for 10 minutes.
1.3.8. Decant isopropanol.
1.3.9. Add 1 .0 ml isopropanol to each tube.
1.3.10. Place on the rotator for 5 minutes.
1.3.11. Decant isopropanol.
1.3.12. Place tubes into the speed vac evaporator to dry the solvent. (60 minutes – overnight).
1.3.13. Weigh the tube with hair again and record and calculate net hair weight after wash**,**

1.4. Grinding

As heat generated by the grinding process can cause damage to the sample, it is recommended that the samples be added to the tube containing the beads and stored for 12 hours at low temperatures (below -60), to achieve the best results.

1.4.1. Place the sample into the prefilled grinding tube with zirconium beads.
1.4.2. Remove the bead blaster rotor cover and place tubes in the rotor.

- 1.4.3. Replace cover.
- 1.4.4. Set speed, time, and cycle on the machine.
  - 1.4.4.1. Speed 6.5
  - 1.4.4.2. Cycle 3
  - 1.4.4.3. Time:30
  - 1.4.4.4. Intermission:30
- 1.4.5. Grind hair into powder.

1.5. Cortisol Extraction
- 1.5.1. Add 1.0 ml methanol to each tube.
- 1.5.2. Put the tube on a rotator with constant rotating for 24 hours.
- 1.5.3. Centrifuge the tubes at 14,000 rpm for 5 minutes.
- 1.5.4. Transfer 0.5 ml of the supernatant to a new 1.7 ml Eppendorf tube.
- 1.5.5. Evaporate solvent – place in evaporator in concentrator for at least 90 minutes.

Drug Use Urine. Participants will provide urine samples for on-site toxicology screening using an iCup® test kit (Redwood Toxicology Laboratory, Inc.). Urine screening kits are tested for recent cocaine, methamphetamine, marijuana, opiate, and benzodiazepine use. Urine toxicology screening will serve as a check that can also enhance the accuracy of self-reported drug use.[103,104] Our team has experience collecting drug use data with urine toxicology screening.[76]

For Drug Use Urine toxicology measurement, a urine sample will be collected from the adolescent at each time point. Both the adolescent and the research assessor shall be present (at the same time) during the procedures outlined in this section. The procedures for the data to be stored or associated with each specimen are described below:

1.1 Collect Urine Specimen
- 1.1.1 Remove the cap from the urine cup.
- 1.1.2 Donor provides urine specimen in the collection cup.
- 1.1.3 Fill the cup to at least 1/3 full.
- 1.1.4 Technician replaces and secures the cap while the cup is on a flat surface.
- 1.1.5 Temperature is read at 2-4 minutes.
- 1.1.6 Verify the range is between 90-100 ° F.
- 1.1.7 The collector/technician dates and initials the security seal.
- 1.1.8 Attach the security seal over the cup cap.
- 1.1.9 Transport the cup to the biobehavioral lab.
- 1.1.10 Record the sample drop off in the laboratory sample drop off binder located on top of the small black refrigerator located in the anteroom of 340.

1.2 Reading Results
- 1.2.1 Peel off the label to reveal drug test strips.
- 1.2.2 Peel off label to reveal adulteration strips, if applicable.

1.2.2.1 For cups with Adulteration
1.2.2.1.1 Read adulteration test results between 2-5 minutes.
1.2.2.1.2 Compare the colors on the adulteration strip to the color chart.
1.2.2.1.3 If the results indicate adulteration, do not read the drug test results.
1.2.3 Read the drug test results at 5 minutes. (Do not interpret results after 60 minutes, as false results may occur.

7) **Data Management***

Data Management & Quality Control Procedures. Assessment data will be entered directly by participants onto tablets via REDCap, which is linked to secure university servers. We will follow procedures to ensure quality control in the collection, verification, and documentation of data that we have established previously. Data files will be exported from REDCap and prepared for analysis with statistical software, including M*plus*.

Data Preparation & Preliminary Analyses. Testing of distributional assumptions will include statistical tests for univariate and multivariate normality (tests of skew & kurtosis) as well as visual inspections of the empirical distributions of the data at each time point. Should deviations be deemed sufficient for concern, transformation of variables will be attempted where possible. Reliability estimates of internal consistency (Cronbach's alpha) will be generated for all scale scores. If reliability estimates are found to be below .80, item total correlations and factor analyses will be employed to diagnose and correct psychometric problems.[105]

Measurement Modeling. A family functioning latent construct will be created consisting of two indicators (i.e., parental involvement, and communication) to create a measurement model based on a confirmatory factor analysis using SEM techniques.[106,107] Given that only two indicators will be used to create the latent family functioning construct, we will constrain the factor loadings to be 1 for model identification.[108] In our previous studies, we have consistently found large correlations ($r > .6$) between these two indicators.

AIM 1: Examine the efficacy of FcO, compared to community practice, in decreasing past 90-day drug use (frequency and quantity) and depressive symptoms among HSMY, over 30-months.

This hypothesis will be analyzed using Latent Growth Modeling (LGM).[109] Growth models can be expressed in terms that are identical to models using random coefficients regression, mixed models, or multilevel models, where the first level fits each response in time to an individual-level growth model. In this latter representation the first level fits each response in time to an individual-level growth model and the second level represents how the slope of outcomes (drug use and depressive symptoms) varies by condition. The second level represents the condition effect on the slope of outcomes (drug use and depressive symptoms). There are several advantages to using LGM when investigating

the trajectory of change. First, LGM handles incomplete data, and hence all participants are retained in the analyses. Second, LGM easily handles unequally spaced time intervals. Third, its power is often much greater than ANOVA methods because the unit of analysis is the slope trajectory, and we have 4 time points to measure the growth trajectory of outcomes. Should nonlinearity be found using methods we published,[110,111] we will transform towards linearity.[112] LGM analyses will test for differences in trajectories of outcomes over the 4 time points among the 2 conditions. As recommended by Raudenbush and Bryk,[113] we will use 2-level analysis to determine whether the mean trajectories of outcomes for Familias con Orgullo and control differ significantly over time. To account the non-independence of outcomes, we will conduct multivariate growth modeling similar to that used in evaluating a previous trial.[114] If correlations have above moderate effects ($.30 < r$),[115] we will empirically examine the distinct contributions of the intervention on each separate outcome, using false discovery rate to account for multiple comparisons. Data analysis for this hypothesis and all others will be conducted using M*plus* (v 8.8).[116]

AIM 2: Examine whether the relationship between intervention condition and the outcomes are partially mediated by parent support for the adolescent, parent acceptance, family functioning, adolescent stress, and sexual minority stress (e.g., discrimination).
To test for mediation, we will use the "product of coefficients" test described by MacKinnon, which is based on the distribution of the indirect effect of the intervention through the mediator.[119] This procedure tests whether the product of the coefficients from the intervention to the mediator (a) and from the mediator to the outcome (b) is significantly different from zero. Specifically, we will estimate paths between the intervention condition and mediators (a path), and between mediators and outcomes (b path). Because we have four time points, our first mediation analyses will fit bivariate growth curves for both mediators and outcomes and examine the role of the (latent) slope of mediators on the slope of outcomes. The product of the two pathways (path 'a')*(path 'b') is the indirect effect of FcO to each of the outcome trajectories through the mediators. As noted above, we will be testing whether the product (a)*(b) is statistically significantly different from zero, by comparing the observed value of (a)*(b) to the empirical distribution calculated using the bias-corrected bootstrap.[116] In addition to the mediation models, we will also test if the initial changes in mediators from baseline to the 6 month follow-up mediate the effect of intervention condition on each of the outcome trajectories. Our team has extensive expertise in conducting mediational analyses in multilevel models.[9,12]

Exploratory AIM 3: Explore whether gender and baseline levels of parent support for the adolescent, parent acceptance, family functioning, adolescent resiliency, adolescent stress, and sexual minority stress moderate intervention effects on the youth outcomes.

To test whether gender and baseline levels of parent support for the adolescent, parent acceptance, family functioning, and sexual minority stress moderate intervention effects on the outcomes, an interaction term between intervention condition and each of the potential moderators will be created and entered into a growth curve model by regressing the trajectory slope on condition, gender, and the potential baseline moderators, and their

interaction. For probing the interaction effect, the Johnson-Neyman (JN) technique[121] will be used to assess where moderation occurred. The JN technique identifies regions of significance of intervention effects on the outcome trajectory. Based on the results of the JN technique, we will estimate two separate trajectory models to examine the intervention effects on each of the outcome trajectories.

Post-hoc Analyses. Because we will measure past 90-day frequency and quantity of alcohol and cigarette use, we will examine condition effects on these outcomes. We will also examine intervention effects on sexual risk behaviors (i.e., condomless sex, number of sexual partners, and sex while under the influence of drugs and/or alcohol). As such, we will analyze youth differences in outcomes by these subgroups as well as differences in the mediation pathways. Using the same LGM approach described, we will also explore intervention effects on secondary outcomes of past 90-day frequency and quantity of alcohol use and cigarette use as well as condomless sex (as applicable), number of sexual partners, and sex while under the influence of drugs and/or alcohol. Across all analyses, to account biased estimation of intervention effects influenced by low base rates, we will adjust standard errors of intervention effects on the slope of outcomes by using a Huber-White 'sandwich' estimator.[122] We also recognize that there may be subgroups of HSMY such as US born, acculturated Hispanic youth, and low SES families who may be at higher risk for drug use and depression symptoms. We will explore such differences.

8) **Risks to Subjects***

We do not expect that there will be any negative consequences from participating in this study. However, the sensitive nature of some intervention topics (e.g., sexual behaviors and drug use) may be embarrassing and/or distressing to some participants. It is also possible that they may feel tired after answering the questionnaires or made uncomfortable by the questions. Adolescents that are positive for drug use will be evaluated by the study facilitators to determine the need for treatment and referral. At each of the assessments, we will provide resources for where youth can seek services for drug treatment.

Additionally, adolescents that report active thoughts of suicide or attempts, will be given a risk assessment to determine the need for referral and/or treatment.

Video session recordings will be saved onto secure university servers. All data with identifiable information such as names, date of birth, etc., will be numerically labeled and kept separate from assessment data. Only PIs and authorized study personnel will have access to data files and participant linking lists. The risks associated with gathering information from participants by properly trained and supervised professional research staff are low and include risks of loss of privacy and transient psychological distress. The research team has extensive experience in conducting intervention and assessment protocols that involve underserved populations as well as highly confidential personal information. Quality assurance protocols, emergency procedures, and crisis intervention,

and referral procedures have been established in previous trials and can be modified as needed for the proposed study.

9) Potential Benefits to Subjects*

There may be direct benefits to participating in this study. Adolescents may see a decrease in drug use and depressive symptoms compared to community practices. Also, adolescents may see outcomes mediated by changes in parental support, acceptance, family functioning, their own stress as well as sexual minority stress.

10) Vulnerable Populations*

The research involves children under the age of 18. To avoid coercion or undue influence, the adolescents will assent and complete assessments separate from their primary care giver. In case the adolescent does not want to assent, the family will be told they did not meet one or more of the inclusion/exclusion criteria but the facilitator will not reveal to the parent that the child did not assent (to avoid any consequences to the child).

11) Setting

Recruitment will be conducted in collaboration with community agencies that serve sexual minority youth and through referrals from participants. We will also recruit participants from social events attended by sexual minorities, paper flyers posted at community agencies and events, social media, electronic flyers posted on community partners' websites, and through word of mouth. For now, the agencies will include: Alliance for LGBTQ Youth, Counsel and Connect, Inc., Miami-Dade County Public Schools (once approval is received), PFLAG, Pridelines, SF Health Collaborative, SunServe, and Yes Institute. Members of the research team will be at the specified community agencies, to post the recruitment flyer through their websites and social media outlets. We will also post flyers in public spaces and in businesses that grant permission to do so. We will also contact youth who have participated in our prior studies and have agreed to be contacted for future studies. Moreover, we will recruit participants through media platforms including University of Miami media platforms.

12) Resources Available

Dr. Guillermo (Willy) Prado is Professor of Nursing and Health Studies, Public Health Sciences, and Psychology at the University of Miami (UM). Prado has been conducting family intervention research with Hispanic youth for 18 years and has published over 170 peer-reviewed publications in this area, including with Hispanic sexual minority youth.[1,7,8,15,16,75] Prado's research has been recognized by organizations such as the Society for Prevention Research and the Society for Research on Adolescence. Most recently, he was elected to the National Academy of Medicine for his contributions to developing, evaluating, and disseminating evidence-based interventions for Hispanic youth. Prado has received over $75 million of extramural funding as either PI, or Co-I. He has substantial experience managing the research activities of major randomized

clinical trials including recruitment, data collection, quality control, data analysis, and dissemination of study findings. He is immediate past president of the Society for Prevention Research.

Dr. C. Henricks Brown is Professor in the Departments of Psychiatry and Behavioral Sciences, Preventive Medicine and Medical Social Sciences at the Northwestern University, Feinberg School of Medicine and holds an adjunct faculty appointment at the University of Miami. His work has focused on the prevention of drug abuse, conduct disorder, depression, and health equity. Specifically, Dr. Brown has published extensively on new methodology for producing generalized knowledge about drug use and mental health promotion interventions, including developing innovative research designs for effectiveness, and conducting mediational analyses. Dr. Brown is currently the PI of the NIH P30 "Center for Prevention Implementation Methodology for Drug Abuse and HIV," and PI of a NIMH data synthesis project evaluating impact of prevention programs on sexual and gender minority adolescents. He is also the director of the Prevention Science and Methodology Group, a national network of methodologists working on the design of preventive field intervention trials and their analysis. Dr. Brown has been on multiple of Prado's grants as the methodologist and biostatistician and have published extensively together.

Other personnel include Dr. Yannine Estrada, Project Director for the study and an Assistant Scientist at the University of Miami School of Nursing and Health Studies. Estrada, a licensed counseling psychologist, has extensive experience in directing randomized controlled trials. In fact, she has been responsible for directing the four most recently completed studies on Familias Unidas studies and has been responsible for overseeing all aspects of data collection. Estrada also led, in conjunction with Prado, the development of eHealth Familias Unidas and is, therefore, well versed in the technological logistics, background informatics, and process of the intervention, which will be crucial for executing modifications. Estrada has a long track record of working with Prado which extends over 10 years. For this study, Estrada will collaborate with the senior investigative team in the adaptation of FcO along with planning the outcome analyses and preparing manuscripts for publication. Estrada will also be responsible for coordinating the research and research-clinical interface aspects of the study.

Ms. Maria Tapia, Clinical Trainer and Supervisor is a Licensed Clinical Social Worker with over 20 years of experience working with Hispanic families. She is trained in delivering family-based interventions, including eHealth Familias Unidas, to Hispanic parents and their youth. Tapia has engaged over 80% of Hispanic parents into the Familias Unidas intervention over the past 15 years. She also has extensive experience training and supervising the Familias Unidas model. For the proposed study, Tapia will be responsible for training and supervising the primary care facilitators in the delivery of the intervention. She will also conduct the supervision meetings with the facilitators and will help them problem solve potential barriers of intervention engagement and retention. Additionally, Tapia will handle any adverse or serious adverse events that may occur during the intervention sessions.

Assessors will be responsible for screening potential study participants and conducting the parent and youth assessments over the four years. Assessors will conduct over 306 assessments over the 30 months. They will also be responsible for scheduling these assessments. Finally, assessors will also be responsible for maintaining cordial contact in between assessment timepoints, in order to maximize retention rates at assessments.

Other study personnel, such as student, research assistants and facilitators will be trained in study operating procedures, including consent/assent. All study personnel members are CITI trained and are adequately informed about the protocol, research procedures, and their duties and functions. Trainings will be held for study personnel members before the beginning of the study in: protocol, research procedures, and assessments. Study personnel will be asked to role play procedures at the training. If more training is needed, they will be trained again throughout the study. All of the study key personnel have been involved in NIH funded studies and are all familiar with the recruitment/study procedures as those being proposed. Regardless of experience, trainings will still be held by the lead clinical supervisor, project director, and principal investigators to assure all study personnel members are adherent to the study procedures. Weekly meetings will also be held to discuss study updates, issues or concerns.

13) **Prior Approvals**

None.

14) **Recruitment Methods**

To protect participants' sexual minority status, our screening measure also includes questions on levels of physical activity, family communication, quality diet, and substance use. Further, we do not explicitly state that our study has an HSMY focus because qualifying for the study would then identify the status of someone who may not want their sexual minority status revealed. The screening measure is administered via iPads and after the potential participant completes it, the participant will receive a message stating, "you are eligible for this study" or "you are not eligible for this study."

After informed consent/assent, parent and youth dyads complete a 45 minute to 1-hour baseline assessment and then are randomized to either FcO, the experimental condition, or the control condition. We will reconsent youth who turn 18 years of age over the course of the study, as we have done in past studies. Specifically, we will track youth age via a secure database system that will alert the study team when a participant has turned eighteen.

Participants will be assessed at four repeated measure assessments. They are baseline, 6 months, 18 months, and 30 months post-baseline. We have budgeted the following amounts for participants parent incentives: T1=$55, T2=$60, T3 = $65, T4= $70. Adolescents will receive: T1=$20, T2=$25, T3 = $30, T4= $35 for their assessments.

The participant incentive cost will reimburse study participants for time and effort to participate in the baseline and each of the follow-up assessment activities.

15) **Confidentiality**

The issues surrounding confidentiality are of supreme importance and sensitivity because personal information will be obtained from the adolescent and their family members. Participants sign a statement attesting to their understanding that the information they provide will be held as personal and confidential. Consent forms clearly state the right to refuse participation at any time. In addition, because this project involves the collection of mental health, drug use and sexual behavior information, to strengthen the security of our records in relation to local and state courts, a Confidentiality Certificate under section 502C of Part E, Title II of the Comprehensive Drug Abuse Prevention and Control Act of 1970, Public Law 91-513, has been obtained. This Certificate has been utilized in previous prevention studies and has been instrumental in maintaining the privacy/confidentiality of sensitive client information. With this Certificate, the researchers cannot be forced, even by a court order, to share research information that may identify the participants in any civil, criminal, administrative, legislative, or other proceedings in any court. The researchers will use the Certificate to resist any demands for information that would identify the participants, except to prevent serious harm to them or others, and as explained below.

The participants should understand that a Certificate of Confidentiality does not prevent them, or a member of their family, from voluntarily releasing information about themselves, or their involvement in this study. If an insurer or employer learns about their participation, and obtains participant's consent to receive research information, then we may not use the Certificate of Confidentiality to withhold this information. This means that the participants and their family must also actively protect their own privacy. Disclosure will be necessary, however, upon request for the purpose of audit or evaluation, and is limited only to Department of Health and Human Services employees involved in the review. The participant should understand that we will in all cases, take the necessary action, which may include reporting to authorities, to prevent serious harm to themselves or others.

15(b). Data collected:

☐ Will not include Protected Health information or Personally Identifiable Information

☒ Will include Protected Health information or Personally Identifiable Information

15(c). How will the research store the data?

☐ On a University of Miami electronic device (e.g. encrypted, password-protected computer)

☒ On a cloud-based storage system that is approved by the University of Miami

☐ Other, specify: Click here to enter text.

Select one of the following:

☐ The Principal Investigator (and/or Study Team members) will record (e.g. write down, abstract) data acquired in a manner that **does not include any** indirect or direct identifiers (listed in the instructions for Section 15 of this protocol), and the recorded data will not be linked to the individual's' identity.

OR

☒ The Principal investigator (and/or Study Team members) will record (e.g. write down, abstract) the data collected in a manner that does not include any direct identifiers (see list in the instructions for Section 15 of this protocol) of any subject. Instead, the Principal Investigator and/or Study Team members shall will assign a code (that is not derived in whole or in part from any direct or indirect identifiers of the individual) to each study subject and link the code to the study subject's identity. **The link to each subject's identity and/ or other identifiable information will be maintained on a document separate from the research data.**

Biospecimens

☐ Not applicable. No biospecimens will be collected

☐ *Bio*-Specimens obtained for this research will be stored without any direct or indirect identifiers.

☐ *Bio*-Specimens obtained for this research will be stored in a de-identified coded manner.

☒ **-** When required to transport data or bio-specimens for this research, the research team will transport the data and bio-specimens in a de-identified (or anonymous) manner with a link to the individual subject's identity maintain separately from the data and/or bio-specimen.

15d. Jackson Health System additional requirement

☒ **This section is not applicable because the research is not collecting health information from JHS under a waiver of authorization (without obtaining a HIPAA authorization from the participant)**

If health information, including Protected Health Information and/or Personally Identifiable Information are collected from JHS without a signed authorization from the subject (with a waiver of authorization from an IRB or Privacy Board), you must agree to the following:

☐

JHS data, including Protected Health Information (PHI) and/or Personally Identifiable Information (PII), acquired from JHS for this research with a waiver of the requirement for an authorization under HIPAA shall only be stored on the secured JHS SharePoint environment made available by JHS. I and the Study Team members shall not copy or store the JHS sourced personally identifiable information (PII), including protected health information (PHI) data to any other system, including any systems maintained or provided by the University of Miami. I and the Study Team shall only copy or transfer JHS-sourced data that has been properly de-identified in accordance with all requirements contained in the HIPAA Rules by removing all of the identifiers listed in the instructions for Section 15 of this protocol.

If the data obtained for this research will be acquired from a retrospective "chart review" involving health information from JHS with a waiver of authorization (without obtaining an signed HIPAA authorization from the subject) then the data and the link and/or key to each subject's identity shall only be maintained in the secure JHS SharePoint environment made available by JHS.

16) Provisions to Protect the Privacy Interests of Subjects

During the recruitment and electronic consent/assent process, and prior to the assessments, participants are told that information is private, confidential and only accessed by specific study personnel. Participants are given a case ID to further protect their identity. Participants are free to ask any questions, and the consent form has the contact information of the Principal Investigator should they have further questions and the phone number to the University of Miami's Human Subjects office. In the informed consent forms, participants will be advised that we may use deidentified survey data collected for research purposes and provide the de-identified survey data to other researchers for further analyses. Participants' information will be removed from the survey data. Once the identifiers have been removed, we will not ask for participants' consent to use or share their survey data for research. We do not expect for this study to create more than minimal risk for the participating families. Yet, the study will be monitored by the study investigators and/or sponsors.

17) Waiver of Authorization for Use and Disclosure of Protected Health Information (HIPAA)

☒ This section is not applicable, we are not requesting a waiver of authorization.

If the research team will access patient medical records or other identifiable health information for this research without or prior to obtaining a signed HIPAA authorization from the subject or the subject's legally authorized representative (LAR), you must obtain a waiver of the requirement for written authorization from the patients to access their medical records.

Confirm that you will destroy the Protected Health Information (PHI) you and/or your Study Team acquire receive from JHS and/or UHealth at the earliest opportunity.

☐ ***I confirm***

Confirm that the Protected Health Inform (PHI) you acquire from JHS and/or UHealth will not be re-used or disclosed to any other person or entity, except as required by law or for authorized oversight of the research study or for other research for which the use or disclosure of PHI is permissible.

☐ ***I confirm***

If you are collecting health information from JHS under a waiver of authorization, you must read the paragraph below and sign the signature block to indicate your agreement:

Notwithstanding the preceding "I confirm" statements above, I agree that neither I nor any member of the study team listed on the IRB submission for this Protocol shall ever re-use or re-disclose any of the information acquired from Jackson Health System in any format, whether **identifiable or de-identified,** to any individual or entity without first obtaining written permission from Jackson Health System, even if such re-use or re-disclosure is permissible by law (e.g., HIPAA).

____

PI Signature Date

18) Consent Process

The consent process will take place on tablets. A study team member will present and read the electronic consent forms to the caregivers who are eligible to participate based on the inclusion and exclusion criteria. The consent form will be an electronic document bearing the HSRO approval stamp/watermark. The consent and assents will be available in English or Spanish. We will use the same procedures for translating and back translating as in previous studies. After the

caregivers understand the study and after all questions have been answered to their satisfaction, individuals who elect to participate in the study will be asked to sign the consent form. The consent process will last approximately 30 minutes, but it may take caregivers a little less or a little more depending on how clearly they comprehend the study procedures.

If a parent is not available to complete consent, a legal guardian is allowed to provide permission for the adolescent to assent. A legal guardian is a step mother or step father, or another person who has obtained legal rights to the child. During the recruitment screening process, study personnel will determine if the primary caregiver is a legal guardian if they demonstrate the correct paperwork.

If other family members (adults or minors) want to participate in the virtual family sessions. They will be required to sign an electronic secondary informed consent through REDCap. (1) For other adults, **the secondary adult consent**. (2) For minor(s) to have signed, from the parent or legal guardian, **a secondary adult consent for a minor**, and the **secondary minor assent for the other minor**(s).

19) Process to Document Consent in Writing

Consents will be stored electronically in secure University of Miami servers. Information for de-identified participants who do not wish to participate in the study and provided verbal consent/assent to answer the questions for non-participating families will be kept in secure University of Miami servers.

References

1. Ocasio MA, Feaster DJ, Prado G. Substance use and sexual risk behavior in sexual minority Hispanic adolescents. *Journal of Adolescent Health.* 2016;59(5):599-601.
2. Kann L, Olsen EO, McManus T, et al. Sexual Identity, Sex of Sexual Contacts, and Health-Related Behaviors Among Students in Grades 9-12 - United States and Selected Sites, 2015. *MMWR Surveill Summ.* 2016;65(9):1-202.
3. Ryan C, Huebner D, Diaz RM, Sanchez J. Family rejection as a predictor of negative health outcomes in white and Latino lesbian, gay, and bisexual young adults. *Pediatrics.* 2009;123(1):346-352.
4. Simons L, Schrager SM, Clark LF, Belzer M, Olson J. Parental support and mental health among transgender adolescents. *Journal of Adolescent Health.* 2013;53(6):791-793.
5. Ryan C, Russell ST, Huebner D, Diaz R, Sanchez J. Family acceptance in adolescence and the health of LGBT young adults. *Journal of Child and Adolescent Psychiatric Nursing.* 2010;23(4):205-213.

6. Chrisler AJ. Understanding Parent Reactions to Coming Out as Lesbian, Gay, or Bisexual: A Theoretical Framework. *Journal of Family Theory & Review.* 2017;9(2):165-181.
7. Estrada Y, Lozano A, Tapia M, I., et al. *Familias con Orgullo*: Pilot study of a family intervention for Latinx sexual minority youth. Under Review.
8. Lozano A, Estrada Y, Tapia MI, et al. Development of a family-based preventive intervention for Latinx sexual minority youth and their parents. *Cultural diversity and ethnic minority psychology.* 2021.
9. Estrada Y, Lee TK, Huang S, et al. Parent-Centered Prevention of Risky Behaviors Among Hispanic Youths in Florida. *Am J Public Health.* 2017;107(4):607-613.
10. Estrada Y, Rosen A, Huang S, et al. Efficacy of a brief intervention to reduce substance use and human immunodeficiency virus infection risk among Latino youth. *Journal of Adolescent Health.* 2015;57(6):651-657.
11. Prado G, Cordova D, Huang S, et al. The efficacy of Familias Unidas on drug and alcohol outcomes for Hispanic delinquent youth: Main effects and interaction effects by parental stress and social support. *Drug and alcohol dependence.* 2012;125:S18-S25.
12. Prado G, Pantin H, Briones E, et al. A randomized controlled trial of a parent-centered intervention in preventing substance use and HIV risk behaviors in Hispanic adolescents. *Journal of Consulting and Clinical Psychology.* 2007;75(6):914-926.
13. Prado G, Pantin H, Huang S, et al. Effects of a family intervention in reducing HIV risk behaviors among high-risk Hispanic adolescents: A randomized controlled trial. *Archives of pediatrics & adolescent medicine.* 2012;166(2):127-133.
14. Pantin H, Prado G, Lopez B, et al. A randomized controlled trial of Familias Unidas for Hispanic adolescents with behavior problems. *Psychosomatic medicine.* 2009;71(9):987.
15. Ocasio MA, Lozano A, Lee TK, Feaster DJ, Carrico A, Prado G. Efficacy of a Family-Based Intervention for HIV Prevention with Hispanic Adolescents with Same Gender Sexual Behaviors. *Prevention Science.* 2021.
16. Lozano A, Fernández A, Tapia MI, Estrada Y, Juan Martinuzzi L, Prado G. Understanding the Lived Experiences of Hispanic Sexual Minority Youth and their Parents. *Fam Process.* 2021.
17. Perrino T, Pantin H, Prado G, et al. Preventing internalizing symptoms among Hispanic adolescents: A synthesis across Familias Unidas trials. *Prevention Science.* 2014;15(6):917-928.
18. Baiden P, LaBrenz C, Asiedua-Baiden G, Muehlenkamp JJ. Examining the intersection of race/ethnicity and sexual orientation on suicidal ideation and suicide attempt among adolescents: Findings from the 2017 Youth Risk Behavior Survey. *Journal of psychiatric research.* 2020.
19. Lewis NM, Wilson K. HIV risk behaviours among immigrant and ethnic minority gay and bisexual men in North America and Europe: A systematic review. *Social Science & Medicine.* 2017;179:115-128.
20. Luk JW, Gilman SE, Haynie DL, Simons-Morton BG. Sexual orientation and depressive symptoms in adolescents. *Pediatrics.* 2018;141(5):e20173309.

21. Shearer A, Herres J, Kodish T, et al. Differences in Mental Health Symptoms Across Lesbian, Gay, Bisexual, and Questioning Youth in Primary Care Settings. *J Adolesc Health.* 2016;59(1):38-43.
22. Stone DM, Luo F, Ouyang L, Lippy C, Hertz MF, Crosby AE. Sexual orientation and suicide ideation, plans, attempts, and medically serious attempts: Evidence from local youth risk behavior surveys, 2001–2009. *American journal of public health.* 2014;104(2):262-271.
23. Marshal MP, Dietz LJ, Friedman MS, et al. Suicidality and depression disparities between sexual minority and heterosexual youth: a meta-analytic review. *J Adolesc Health.* 2011;49(2):115-123.
24. Math SB, Seshadri SP. The invisible ones: sexual minorities. *Indian J Med Res.* 2013;137(1):4-6.
25. D'Augelli AR, Grossman AH, Starks MT. Parents' awareness of lesbian, gay, and bisexual youths' sexual orientation. *Journal of Marriage and Family.* 2005;67(2):474-482.
26. Hobaica S, Alman A, Jackowich S, Kwon P. Empirically based psychological interventions with sexual minority youth: a systematic review. *Psychology of Sexual Orientation and Gender Diversity.* 2018;5(3):313.
27. Newcomb ME, LaSala MC, Bouris A, et al. The influence of families on LGBTQ youth health: A call to action for innovation in research and intervention development. *LGBT health.* 2019;6(4):139-145.
28. Johns MM, Liddon N, Jayne PE, Beltran O, Steiner RJ, Morris E. Systematic mapping of relationship-level protective factors and sexual health outcomes among sexual minority youth: The role of peers, parents, partners, and providers. *LGBT health.* 2018;5(1):6-32.
29. O'Connell M, Boat T, Warner K. National Research Council (US) and Institute of Medicine (US) Committee on the Prevention of Mental Disorders and Substance Abuse Among Children, Youth and Young Adults. Preventing Mental, Emotional, and Behavioral Disorders Among Young People: Progress and Possibilities. *Preventing mental, emotional, and behavioral disorders among young people: Progress and possibilities.* 2009.
30. Dishion TJ, Stormshak EA. *Intervening in children's lives: An ecological, family-centered approach to mental health care.* American Psychological Association; 2007.
31. Brody GH, Chen Y-F, Kogan SM, Murry VM, Brown ACJJoc, psychology c. Long-term effects of the strong African American families program on youths' alcohol use. 2010;78(2):281.
32. Hentges RF, Krug CMW, Shaw DS, Wilson MN, Dishion TJ, Lemery-Chalfant K. The long-term indirect effect of the early Family Check-Up intervention on adolescent internalizing and externalizing symptoms via inhibitory control. *Development and psychopathology.* 2020;32(4):1544-1554.
33. Singer M, Bulled N, Ostrach B, Mendenhall E. Syndemics and the biosocial conception of health. *The Lancet.* 2017;389(10072):941-950.
34. Singer MC, Erickson PI, Badiane L, et al. Syndemics, sex and the city: understanding sexually transmitted diseases in social and cultural context. *Social science & medicine.* 2006;63(8):2010-2021.

35. Singer M. AIDS and the health crisis of the US urban poor; the perspective of critical medical anthropology. *Social science & medicine.* 1994;39(7):931-948.
36. Singer M. *Introduction to syndemics: A critical systems approach to public and community health.* John Wiley & Sons; 2009.
37. Stall R, Friedman M, Catania JA. Interacting epidemics and gay men's health: a theory of syndemic production among urban gay men. *Unequal opportunity: Health disparities affecting gay and bisexual men in the United States.* 2008;1:251-274.
38. Mizuno Y, Borkowf C, Millett GA, Bingham T, Ayala G, Stueve A. Homophobia and racism experienced by Latino men who have sex with men in the United States: correlates of exposure and associations with HIV risk behaviors. *AIDS and Behavior.* 2012;16(3):724-735.
39. Martinez O, Arreola S, Wu E, et al. Syndemic factors associated with adult sexual HIV risk behaviors in a sample of Latino men who have sex with men in New York City. *Drug and alcohol dependence.* 2016;166:258-262.
40. Mustanski B, Garofalo R, Herrick A, Donenberg G. Psychosocial health problems increase risk for HIV among urban young men who have sex with men: preliminary evidence of a syndemic in need of attention. *Annals of Behavioral Medicine.* 2007;34(1):37-45.
41. Stall R, Mills TC, Williamson J, et al. Association of co-occurring psychosocial health problems and increased vulnerability to HIV/AIDS among urban men who have sex with men. *American journal of public health.* 2003;93(6):939-942.
42. Newcomb ME, Feinstein BA, Matson M, Macapagal K, Mustanski B. "I Have No Idea What's Going On Out There:" Parents' Perspectives on Promoting Sexual Health in Lesbian, Gay, Bisexual, and Transgender Adolescents. *Sexuality Research and Social Policy.* 2018;15(2):111-122.
43. Russell ST, Fish JN. Mental Health in Lesbian, Gay, Bisexual, and Transgender (LGBT) Youth. *Annu Rev Clin Psychol.* 2016;12:465-487.
44. Przeworski A, Piedra A. The role of the family for sexual minority Latinx individuals: A systematic review and recommendations for clinical practice. *Journal of GLBT Family Studies.* 2020;16(2):211-240.
45. McConnell EA, Birkett M, Mustanski B. Families Matter: Social Support and Mental Health Trajectories Among Lesbian, Gay, Bisexual, and Transgender Youth. *J Adolesc Health.* 2016;59(6):674-680.
46. Snapp SD, Watson RJ, Russell ST, Diaz RM, Ryan C. Social support networks for LGBT young adults: Low cost strategies for positive adjustment. *Family Relations.* 2015;64(3):420-430.
47. Richter BEJ, Lindahl KM, Malik NM. Examining Ethnic Differences in Parental Rejection of LGB Youth Sexual Identity. *Journal of family psychology : JFP : journal of the Division of Family Psychology of the American Psychological Association.* 2017;31(2):244-249.
48. Mustanski B, Wagenen AV, Birkett M, Eyster S, Corliss HL. Identifying Sexual Orientation Health Disparities in Adolescents: Analysis of Pooled Data From the Youth Risk Behavior Survey, 2005 and 2007. *American Journal of Public Health.* 2014;104(2):211-217.

49. Rothman EF, Sullivan M, Keyes S, Boehmer U. Parents' supportive reactions to sexual orientation disclosure associated with better health: Results from a population-based survey of LGB adults in Massachusetts. *Journal of homosexuality.* 2012;59(2):186-200.
50. Detrie PM, Lease SH. The relation of social support, connectedness, and collective self-esteem to the psychological well-being of lesbian, gay, and bisexual youth. *Journal of Homosexuality.* 2007;53(4):173-199.
51. Pastrana A. Being out to others: The relative importance of family support, identity and religion for LGBT latina/os. *Latino Studies.* 2015;13(1):88-112.
52. Bouris A, Guilamo-Ramos V, Pickard A, et al. A Systematic Review of Parental Influences on the Health and Well-Being of Lesbian, Gay, and Bisexual Youth: Time for a New Public Health Research and Practice Agenda. *The Journal of Primary Prevention.* 2010;31(5):273-309.
53. Katz-Wise SL, Rosario M, Tsappis M. LGBT youth and family acceptance. *Pediatric Clinics of North America.* 2016;63(6):1011.
54. Shilo G, Savaya R. Effects of family and friend support on LGB youths' mental health and sexual orientation milestones. *Family Relations.* 2011;60(3):318-330.
55. Mitrani VB, De Santis JP, McCabe BE, Deleon DA, Gattamorta KA, Leblanc NM. The Impact of Parental Reaction to Sexual Orientation on Depressive Symptoms and Sexual Risk Behavior Among Hispanic Men Who Have Sex with Men. *Arch Psychiatr Nurs.* 2017;31(4):352-358.
56. Finlinson HA, Colón HM, Robles RR, Soto M. An exploratory study of Puerto Rican MSM drug users: The childhood and early teen years of gay males and transsexual females. *Youth & Society.* 2008;39(3):362-384.
57. Wang L. Verbal stigmatization from family: How does that affect Latino men who have sex with men? *Journal of Social and Personal Relationships.* 2019;36(1):3-21.
58. Muñoz-Laboy M, Ripkin A, Garcia J, Severson N. Family and work influences on stress, anxiety and depression among bisexual Latino men in the New York City Metropolitan Area. *Journal of immigrant and minority health.* 2015;17(6):1615-1626.
59. Rosario M, Schrimshaw EW, Hunter J. Predicting different patterns of sexual identity development over time among lesbian, gay, and bisexual youths: a cluster analytic approach. *Am J Community Psychol.* 2008;42(3-4):266-282.
60. Huebner DM, Roche KM, Rith KA. Effects of Family Demographics and the Passage of Time on Parents' Difficulty with Their Lesbian, Gay, or Bisexual Youth's Sexual Orientation. *Archives of sexual behavior.* 2019;48(5):1581-1588.
61. Cauce AM, Domenech-Rodríguez M, Paradise M, et al. Cultural and contextual influences in mental health help seeking: a focus on ethnic minority youth. *Journal of consulting and clinical psychology.* 2002;70(1):44.
62. Dillon FR, Worthington RL, Moradi B. Sexual identity as a universal process. In: *Handbook of identity theory and research.* Springer; 2011:649-670.
63. Hirai M, Winkel MH, Popan JR. The role of machismo in prejudice toward lesbians and gay men: Personality traits as moderators. *Personality and Individual Differences.* 2014;70:105-110.

64. Diaz-Stevens AM. *Recognizing the Latino resurgence in US religion: The Emmaus paradigm.* Routledge; 2018.
65. Gattamorta K, Quidley-Rodriguez N. Coming out experiences of Hispanic sexual minority young adults in South Florida. *Journal of homosexuality.* 2018;65(6):741-765.
66. De Santis JP, Gattamorta KA, Valdes B, Sanchez M, Provencio-Vasquez EJS, Culture. The relationship of Hispanic cultural factors and sexual behaviors of Hispanic men who have sex with men. 2019;23(1):292-309.
67. Austin AM, Macgowan MJ, Wagner EF. Effective family-based interventions for adolescents with substance use problems: A systematic review. *Research on social work practice.* 2005;15(2):67-83.
68. Perrino T, Pantin H, Huang S, Brincks A, Brown CH, Prado G. Reducing the risk of internalizing symptoms among high-risk Hispanic youth through a family intervention: A randomized controlled trial. *Family process.* 2016;55(1):91-106.
69. Prado G, Pantin H. Reducing Substance Use and HIV Health Disparities among Hispanic Youth in the U.S.A.: The Familias Unidas Program of Research. *Intervencion psicosocial.* 2011;20(1):63-73.
70. Coatsworth JD, Santisteban DA, McBride CK, Szapocznik J. Brief Strategic Family Therapy versus community control: engagement, retention, and an exploration of the moderating role of adolescent symptom severity. *Fam Process.* 2001;40(3):313-332.
71. Tapia MI, Schwartz SJ, Prado G, Lopez B, Pantin H. Parent-centered intervention: A practical approach for preventing drug abuse in Hispanic adolescents. *Research on Social Work Practice.* 2006;16(2):146-165.
72. Kuntsche S, Kuntsche E. Parent-based interventions for preventing or reducing adolescent substance use—A systematic literature review. *Clinical psychology review.* 2016;45:89-101.
73. Gibbons RD, Kupfer DJ, Frank E, et al. Computerized Adaptive Tests for Rapid and Accurate Assessment of Psychopathology Dimensions in Youth. *Journal of the American Academy of Child & Adolescent Psychiatry.* 2020;59(11):1264-1273.
74. Gibbons RD, Alegria M, Markle S, et al. Development of a computerized adaptive substance use disorder scale for screening and measurement: the CAT-SUD. *Addiction.* 2020;115(7):1382-1394.
75. Ocasio MA, Tapia GR, Lozano A, Carrico AW, Prado G. Internalizing symptoms and externalizing behaviors in Latinx adolescents with same sex behaviors in Miami. *Journal of LGBT Youth.* 2020:1-17.
76. Carrico AW, Gómez W, Jain J, et al. Randomized controlled trial of a positive affect intervention for methamphetamine users. *Drug Alcohol Depend.* 2018;192:8-15.
77. Carrico AW, Neilands TB, Dilworth SE, et al. Randomized controlled trial of a positive affect intervention to reduce HIV viral load among sexual minority men who use methamphetamine. *J Int AIDS Soc.* 2019;22(12):e25436.
78. Perrino T, Brincks A, Howe G, Brown CH, Prado G, Pantin H. Reducing internalizing symptoms among high-risk, Hispanic adolescents: Mediators of a preventive family intervention. *Prevention Science.* 2016;17(5):595-605.

79. Vidot DC, Huang S, Poma S, Estrada Y, Lee TK, Prado G. Familias Unidas' Crossover Effects on Suicidal Behaviors among Hispanic Adolescents: Results from an Effectiveness Trial. *Suicide and Life-Threatening Behavior.* 2016;46:S8-S14.
80. National Academies of Sciences E, Medicine. *Fostering Healthy Mental, Emotional, and Behavioral Development in Children and Youth: A National Agenda.* National Academies Press; 2019.
81. Fernandez A, Lozano A, Lee TK, Estrada Y, Messiah SE, Prado G. A Family-Based Healthy Lifestyle Intervention: Crossover Effects on Substance Use and Sexual Risk Behaviors. *Prevention Science.* 2021;22(5):602-608.
82. Sandler IN, Schoenfelder EN, Wolchik SA, MacKinnon DP. Long-Term Impact of Prevention Programs to Promote Effective Parenting: Lasting Effects but Uncertain Processes. *Annual Review of Psychology.* 2011;62(1):299-329.
83. Tapia MI, Lozano A, Estrada Y, Fernandez A, Prado G, Austin A. Evaluating Measurement Properties of a Modified Affirmative Counseling Skills Scale with Social Workers and School Psychologists in South Florida. *Journal of Gay & Lesbian Mental Health.* In Press.
84. Szapocznik J, Coatsworth JD. An ecodevelopmental framework for organizing the influences on drug abuse: A developmental model of risk and protection. In: *Drug abuse: Origins & interventions.*: American Psychological Association; 1999:331-366.
85. Meyer IH. Prejudice, social stress, and mental health in lesbian, gay, and bisexual populations: conceptual issues and research evidence. *Psychol Bull.* 2003;129(5):674-697.
86. Szapocznik J, Kurtines WM, Foote FH, Perez-Vidal A, Hervis O. Conjoint versus one-person family therapy: Some evidence for the effectiveness of conducting family therapy through one person. *Journal of Consulting and Clinical Psychology.* 1983;51(6):889.
87. Szapocznik J, Perez-Vidal A, Brickman AL, et al. Engaging adolescent drug abusers and their families in treatment: A strategic structural systems approach. *Journal of Consulting and Clinical Psychology.* 1988;56(4):552.
88. Szapocznik J, Rio A, Murray E, et al. Structural family versus psychodynamic child therapy for problematic Hispanic boys. *Journal of Consulting and Clinical Psychology.* 1989;57(5):571.
89. Burns B, Angold A, Magruder-Habib K, Costello E, Patrick M. Child and Adolescent Services Assessment (CASA) manual. *Durham, NC: Duke University Medical Center, Developmental Epidemiology Program.* 1996.
90. Harris PA, Taylor R, Thielke R, Payne J, Gonzalez N, Conde JG. Research electronic data capture (REDCap)—a metadata-driven methodology and workflow process for providing translational research informatics support. *Journal of biomedical informatics.* 2009;42(2):377-381.
91. Radloff LS. The CES-D scale: A self-report depression scale for research in the general population. *Applied psychological measurement.* 1977;1(3):385-401.
92. Johnston LD, Miech RA, O'Malley PM, Bachman JE, Patrick ME. Monitoring the Future National Survey Results on Drug Use, 1975-2019: Overview, Key Findings on Adolescent Drug Use. *Institute for Social Research.* 2020.

93. Jemmott III JB, Jemmott LS, Fong GT. Abstinence and safer sex HIV risk-reduction interventions for African American adolescents: a randomized controlled trial. *Jama.* 1998;279(19):1529-1536.
94. Gorman-Smith D, Tolan PH, Zelli A, Huesmann LR. The relation of family functioning to violence among inner-city minority youths. *Journal of Family Psychology.* 1996;10(2):115.
95. Barnes HL, Olson DH. Parent-adolescent communication and the circumplex model. *Child development.* 1985:438-447.
96. Zimet GD, Powell SS, Farley GK, Werkman S, Berkoff KA. Psychometric Characteristics of the Multidimensional Scale of Perceived Social Support. *Journal of Personality Assessment.* 1990;55(3-4):610-617.
97. Willoughby BL, Malik NM, Lindahl KM. Parental reactions to their sons' sexual orientation disclosures: The roles of family cohesion, adaptability, and parenting style. *Psychology of Men & Masculinity.* 2006;7(1):14.
98. Goldbach JT, Gibbs JJ. A developmentally informed adaptation of minority stress for sexual minority adolescents. *J Adolesc.* 2017;55:36-50.
99. Cohen S, Kamarck T, Mermelstein R. A global measure of perceived stress. *Journal of health and social behavior.* 1983:385-396.
100. Ryder AG, Alden LE, Paulhus DL. Is acculturation unidimensional or bidimensional? A head-to-head comparison in the prediction of personality, self-identity, and adjustment. *Journal of personality and social psychology.* 2000;79(1):49.
101. Klein F, Sepekoff B, Wolf TJ. Sexual orientation: A multi-variable dynamic process. *Journal of Homosexuality.* 1985;11(1-2):35-49.
102. Musana JW, Cohen CR, Kuppermann M, et al. Association of differential symptoms of stress to hair cortisol and cortisone concentrations among pregnant women in Kenya. *Stress.* 2020;23(5):556-566.
103. Jones EE, Sigall H. The bogus pipeline: a new paradigm for measuring affect and attitude. *Psychological bulletin.* 1971;76(5):349.
104. Roese NJ, Jamieson DW. Twenty years of bogus pipeline research: A critical review and meta-analysis. *Psychological bulletin.* 1993;114(2):363.
105. Nunnally JC. *Psychometric Theory: 2d Ed.* McGraw-Hill; 1978.
106. Bollen KA. Measurement Models: The Relation between Latent and Observed Variables. In: *Structural Equations with Latent Variables.*1989:179-225.
107. Kline RB. Structural equation modeling. *New York: Guilford.* 1998.
108. Kenny D, Kashy D, Cook W. Dyadic data analysis: Guilford press. *United Kingdom.* 2006.
109. Bollen KA, Curran PJ. *Latent curve models: A structural equation perspective.* Vol 467: John Wiley & Sons; 2006.
110. Wang C-P, Hendricks Brown C, Bandeen-Roche K. Residual diagnostics for growth mixture models: Examining the impact of a preventive intervention on multiple trajectories of aggressive behavior. *Journal of the American Statistical Association.* 2005;100(471):1054-1076.
111. Brown CH, Wang W, Kellam SG, et al. Methods for testing theory and evaluating impact in randomized field trials: Intent-to-treat analyses for integrating the

perspectives of person, place, and time. *Drug and alcohol dependence.* 2008;95:S74-S104.
112. Brown CH, Brincks A, Huang S, et al. Two-year impact of prevention programs on adolescent depression: An integrative data analysis approach. *Prevention Science.* 2018;19(1):74-94.
113. Raudenbush SW, Bryk AS. *Hierarchical linear models: Applications and data analysis methods.* Vol 1: sage; 2002.
114. Brown CH, Chamberlain P, Saldana L, Padgett C, Wang W, Cruden G. Evaluation of two implementation strategies in 51 child county public service systems in two states: results of a cluster randomized head-to-head implementation trial. *Implementation Science.* 2014;9(1):1-15.
115. Cohen J. Statistical power analysis for the social sciences. 1988.
116. Muthén LK, Muthen B. *Mplus user's guide: Statistical analysis with latent variables, user's guide.* Muthén & Muthén; 2017.
117. Muthén LK, Muthén BO. How to use a Monte Carlo study to decide on sample size and determine power. *Structural equation modeling.* 2002;9(4):599-620.
118. Sandler I, Ingram A, Wolchik S, Tein JY, Winslow E. Long-term effects of parenting-focused preventive interventions to promote resilience of children and adolescents. *Child Development Perspectives.* 2015;9(3):164-171.
119. MacKinnon DP. *Introduction to statistical mediation analysis.* Routledge; 2008.
120. Thoemmes F, MacKinnon DP, Reiser MR. Power analysis for complex mediational designs using Monte Carlo methods. *Structural Equation Modeling.* 2010;17(3):510-534.
121. Preacher KJ, Rucker DD, Hayes AF. Addressing moderated mediation hypotheses: Theory, methods, and prescriptions. *Multivariate behavioral research.* 2007;42(1):185-227.
122. Freedman DA. On the so-called "Huber sandwich estimator" and "robust standard errors". *The American Statistician.* 2006;60(4):299-302.
