# Supplementary material for: Familias con Orgullo: Study protocol for an efficacy study of a family-based intervention for Hispanic sexual minority youth
Source: PLoS One. 2023 Dec 15;18(12):e0295683. doi: 10.1371/journal.pone.0295683 (PMC10723681; doi:10.1371/journal.pone.0295683)
Supplement: S2 File — (PDF) [file pone.0295683.s003.pdf]

Permission to Take Part in a Human Research Study—SBS (Parent English)
V1

Title of Study: Promoting Health and Reducing Risk among Hispanic Sexual Minority Youth and their Families
Principal Investigator: Guillermo "Willy" Prado, PhD
Department: School of Nursing and Health Studies (SONHS)
Sponsor: NIMHD

You and your adolescent are being asked to take part in a study by the University of Miami, School of Nursing and Health Studies (SONHS). We are asking you because you are Hispanic and have an adolescent between the ages of 13 - 17 years who has disclosed his or her sexual/gender orientation to you.

What is the purpose of the research?

This study is to examine and evaluate a preventive family-based program to help and support families going through the coming out process.

What happens if I say yes, I want to be in this research?
Activities in this study include surveys and intervention, each is described below.

1) ***Surveys***:
   If you agree to be a part of this study, you will be asked to take four surveys at baseline and 6 months, 18 months, and 30 months after baseline, about 45 minutes each. You will be asked questions about your family, adolescent, and about yourself. The topics asked include questions about your adolescent's sexual orientation, when he/she first realized that he/she had feelings or thoughts that he/she may be gay, lesbian, or bisexual, and what was that experience like. We will also ask you about your relationship with your family and your adolescent. We will ask about your mental health and whether you have used drugs and/or alcohol. Your adolescent will be asked about the relationship he or she has with your family and yourself. Your adolescent will also be asked whether he or she uses drugs, alcohol, and/or cigarettes, mental health (including suicide thoughts and/or attempts) and about their sexual behavior. Finally, we will ask your income and you/your adolescent questions about place of birth, time in this country, and age. We will ask your adolescent to provide his/her contact information. You will answer these questions on a tablet. You/your adolescent do not have to answer any questions that you do not want to. If you decide not to answer questions, nothing bad will happen to you. Also, if you want to stop, you may leave the study at any time.

   Survey data may be collected in person or online using REDCap, a secure data collection tool.

2) ***Non-Survey Data Collection:***
   During each assessment, the samples below will be collected:

a. Hair Test: We will collect ~100 strands of hair from your adolescent to measure stress and drug use. The hair will be cut as close to the scalp as possible from the back of the head.

b. Urine Sample: Your adolescent will be asked to provide urine sample for drug testing.

3) ***Intervention:***
You will be randomly assigned to receive the developed program or not to receive it. We cannot control which program your family will be placed in, but every family will have an equal chance of receiving the developed program or community practice as usual. If you are assigned to receive the program, you will be asked to participate in 7 parent group sessions and four family sessions. Your adolescent will not participate in the parent group sessions. Group sessions are approximately two hours, family sessions are about one hour. Additionally, your adolescent will be asked to participate in three adolescent group sessions of approximately 2 hours each. The program will last about three months.

a. The intervention may be delivered in person, online using secure video conferencing technologies, or using phones.

The group sessions and family sessions will be focused on the prevention of drug use, prevention of sexual risk behaviors, improving how families function (e.g., improving the ways parents and youth talk to each other), and parenting skills (e.g., helping parents become aware of peer activities). Additionally, there will be discussion around your adolescent's sexual orientation and the coming out process. In the adolescent groups, adolescents will discuss managing peer pressure and topics regarding being a sexual minority.

Audio/Video Recordings
The intervention sessions will be video recorded. If you do not wish to be videotaped you can sit behind the video camera. If you decline videotaping, your family will not be penalized in any way and will continue to receive the same services. These video recordings will be used for research, teaching, training, and presenting at scientific conferences. Recordings will be uploaded to a protected University of Miami server. All files will be password protected.

What should I think about before I enroll in this research?
Being in this study is up to you. You should ask any questions you may have and get answers before you decide. You being in this study may help in making a program for Hispanic teens. Some of the topics may be uncomfortable or may cause embarrassment.

Do I have to be in this research?
No. This study is voluntary. You do not have to be in this study if you do not want to, and you can leave the study at any time. You will not lose any services, benefits, or rights you would normally have if you choose not to be in the study or if you leave the study early.

Is there any way being in this study could be bad for me?
We do not think that there will be any harm to you or your adolescent for being in this study. However, due to the sensitive nature of some topics, some questions may be embarrassing and/or distressing to some participants. It is also possible that participants may feel tired or fatigued or made uncomfortable by the questions that are asked. The questions you answer may cause some emotional distress. The same thing may happen to your adolescent. You do not have to answer any questions you do not want to answer. In case of any emotional or unresolved clinical issues that may surface, the study personnel may refer you to community organizations that can help you.

What are the benefits to being in this study?
Parents and adolescents may feel better after talking about their personal experiences. Additionally, in discussing the "coming out" process in a safe environment, adolescents and/or families may feel good knowing that their lived experiences may help others. Additionally, this study will give the Hispanic community an opportunity to contribute to the development and evaluation of a prevention program for sexual minority youth. In addition, the results will be disseminated to interested members of the scientific community through presentations and publications, which can potentially inform future research and intervention development.

What about my privacy?
The study is private. Only the researchers and, maybe University of Miami reviewers, will need to see what is learned. Also, to protect your adolescent's privacy, we will not tell you what s/he says in her/his responses. We are not allowed to tell anyone about you or your family.

Also, the staff delivering the group sessions or family sessions will not share the information learned in the group sessions or family sessions with anyone. The information learned by the staff delivering the group sessions or family sessions will be kept completely confidential. The person delivering the group sessions or family sessions will not have access to the surveys. Only the staff from the University of Miami will have access to the surveys.

You should know that if we think there may be child abuse or that a family member will cause harm to him/herself, you, or to others, we will report this concern to the proper authorities. If a report is filed, we will refer you and your family to a mental health center that can help you. We will inform you if your adolescent reports active thoughts of suicide or attempts. However, we will not provide any other information they give during the assessment.

Despite taking all the safety measures listed above, there is risk that your privacy will be broken although we will strive to keep all collected information confidential, we cannot guarantee total privacy.

Your information may be looked at and/or copied for research or regulatory purposes by:

- The sponsor, if any;
- Department of Health and Human Services (DHHS);
- other government agencies;
- other University of Miami employees for audit and/or monitoring purposes; and
- other organizations collaborating in the research

A Certificate of Confidentiality (CoC), issued by the NIH, covers this research. A CoC helps protect your identifiable information.

A CoC protects your private information from all legal proceedings. Unless you assent, information from this research study that identifies you will not be shared outside this research except as described above.

- No one can be forced to share your identifiable information for a lawsuit.
- Your information can't be used as evidence even if there is a court subpoena.

The CoC does not prevent some disclosures.

- The researchers can't refuse requests for information from those funding this research. The National Institute of Health may need information to assess this project.

- You can still share information about yourself. You can also freely discuss your involvement in this research, but this is your choice. The information you share will no longer be protected by the CoC.
- The researchers must disclose things required by law. This includes suspected child abuse and neglect, harm to self or others.

This trial will be registered and may report results on www.ClinicalTrials.gov, a publicly available registry of clinical trials.

Sometimes people in our studies move and we can't find them. Therefore, we will ask you to give us the names and telephone numbers of three people who will always know how to reach you, in case we lose contact with you. We will never share anything you tell us with these people.

Payment

If you agree to be in this research study, we will pay you $55 for the first survey, $60 for the second, $65 for the third, and $70 for the last, for your time and effort. Your child will receive $20 for the first survey, $25 for the second, $30 for the third, and $35 for the last for, his/her time and effort.

Your information (both identifiable and de-identified, as relevant) may be used to create products or to deliver services, including some that may be sold and/or make money for others. If this happens, there are no plans to tell you, or to pay you, or to give any compensation to you or your family. Any data obtained for the purposes of this study become the exclusive property of the University of Miami. The University of Miami may retain, preserve, or dispose of this data for research which may result in commercial applications.

Who can I talk to?

If you have questions, concerns, or complaints, or think the research has hurt you, talk to the research team at (305) 284-2002 with Dr. Guillermo “Willy” Prado, the person leading the research.

This research has been reviewed and approved by an Institutional Review Board (“IRB”). The Human Subject Research Office (HSRO) provides administrative support to the University of Miami’s IRBs. Please call the HSRO at 305-243-3195 if you are a participant in any research being conducted by UM, and:

- Your questions, concerns, or complaints are not being answered by the research team
- You cannot reach the research team
- You want to talk to someone besides the research team
- You have questions about your rights as a research subject
- You want to get information or provide input about this research

PARTICIPANT'S STATEMENT/SIGNATURE

- *I have read this form and the research study has been explained to me.*
- *I have been given the chance to ask questions, and my questions have been answered. If I have more questions, I have been told who to call.*
- *I agree to be in the research study described above.*
- *I will receive a copy of this consent form after I sign it.*

____
Youth Name

____
Printed Name of Parent/Legally Acceptable Representative

____ __________________
Signature of Parent/Legally Acceptable Representative Date

____
Printed Name of Person Obtaining Consent

____ __________________
Signature of Person Obtaining Consent Date

Future Studies

We may conduct additional studies related to the study we are describing in this form. If we do conduct these studies, we may contact you in the future to see if you would like to be part of the future study. Your participation in this study does not depend in any way on your participation in any future study.

- *I agree to be contacted after the study ends to be asked to take part in future studies.*
- *I agree to allow my teen (if he/she is under the age of 18) to be contacted after the study ends to be asked to take part in future studies.*

____
Youth Name

____
Printed Name of Parent/Legally Acceptable Representative

____ __________________
Signature of Parent/Legally Acceptable Representative Date

____
Name of Person Obtaining Consent/Assent

____ __________________
Signature of Person Obtaining Consent/Assent Date
