# Supplementary material for: Familias con Orgullo: Study protocol for an efficacy study of a family-based intervention for Hispanic sexual minority youth
Source: PLoS One. 2023 Dec 15;18(12):e0295683. doi: 10.1371/journal.pone.0295683 (PMC10723681; doi:10.1371/journal.pone.0295683)
Supplement: S3 File — (PDF) [file pone.0295683.s004.pdf]

Permission to Take Part in a Human Research Study—SBS (Adolescent English) V1

Title of Study: Promoting Health and Reducing Risk among Hispanic Sexual Minority Youth and their Families
Principal Investigator: Guillermo "Willy" Prado, PhD
Department: School of Nursing and Health Studies (SONHS)
Sponsor: NIMHD

You are being asked to be in this study because you are Hispanic and have come out to one of your parents. About 306 children will be in the study.

What is the purpose of the research?
This study will help us understand whether a family program can help you and families like yours after the coming out process and to see if you can improve your health. You will need your parent's permission in order to participate.

What happens if I say yes, I want to be in this research?
You will be asked to complete questions and some sessions, each is described below.

1) ***Surveys***:
You will have to complete questions four times: at the beginning and 6 months, 18 months, and 30 months after the beginning, about 45 minutes each. You will be asked questions about your family, parents, and about yourself. Questions are about your sexual orientation, when you first realized that you had feelings or thoughts that you may be gay, lesbian, or bisexual and what was that experience like. We will also ask you about your relationship with your family and your parents. You will also be asked about questions about your behaviors. You do not have to answer any questions that you do not want to. You will answer these questions on a tablet. If you decide not to answer questions, nothing bad will happen to you. Also, if you want to stop, you may leave the study at any time.
Survey data may be collected in person or online.

2) ***Non-Survey Data Collection:***
We will also cut a piece of your hair. This will help us to measure your stress levels. You will also ask us to pee in a cup. This will help us see whether you are using drugs.

3) ***Program sessions:***
You may be asked to be a part of sessions for a program. If you are assigned to receive the program, you will be asked to be in the family sessions. Family sessions are about one hour. Additionally, you will be asked to participate in three adolescent group sessions of approximately 2 hours each (with 12-15 other adolescents). The program will last about three months.
   a. The program will be delivered in person, online using secure video conferencing technologies or using phones

The group sessions and family sessions will be focused on improving how family relations (e.g., improving the ways parents and youth talk to each other). Additionally, we will talk about your sexual orientation and the coming out process.

Audio/Video Recordings
The program sessions will be video recorded. If you do not want to be videotaped, you can sit behind the video camera. If you or your family don't want to be videotaped, nothing bad will happen to you or your family. Recordings are needed to make sure that the person doing the group and family sessions is doing what they are supposed to.

What should I think about before I enroll in this research?
Being in this study is up to you. You should ask any questions you may have and get answers before you decide. You may not like some of the things we talk about.

Do I have to be in this research?
No. You do not have to be in this study if you do not want to, and you can leave the study at any time.

Is there any way being in this study could be bad for me?
We do not think that anything bad will happen to you or your parent for being in this study. However, some questions may make you feel uncomfortable. It is also possible that you may feel tired from answering a lot of questions. You do not have to answer any questions you do not want to answer.

What are the benefits to being in this study?
Parents and adolescents may feel better after talking about their personal experiences. Additionally, in discussing the "coming out" process in a safe environment, adolescents and/or families may feel good knowing that their experiences may help others. In addition, presentations and publications may potentially inform future research and intervention development.

What about my privacy?
The study is private. Only the researchers and, maybe University of Miami reviewers, will need to see what is learned. Also, to protect your privacy, we will not tell your parents what you say. We are not allowed to tell anyone about you or your family. If you test positive for drug use, the research team will conduct an evaluation to determine the need for treatment and referral.

Also, the staff delivering the group sessions or family sessions will not share the information learned in the group sessions or family sessions with anyone. We would also like to let you know that the person delivering the group sessions or family sessions will not have access to the surveys. Only the staff from the University of Miami will have access to the surveys.

You should know that if we think there may be child abuse or that a family member will cause harm to him/herself, you, or to others, we will report this concern to the proper authorities. If a report is filed, we will refer you and your family to a mental health center that can help you. If you report thoughts of suicide or attempts, we will inform your caregiver about this. However, we will not reveal any other information that you report during the surveys. If you report active thoughts of suicide or attempts, we will conduct a risk assessment to determine the need for referral and/or treatment.

There is still a risk that your privacy will be broken although we will strive to keep all collected information confidential, we cannot guarantee total privacy. Facilitators will encourage participants to maintain confidentiality, however we cannot guarantee that your privacy will be maintained. Please remember that while we (the researchers) will keep your information confidential and will remind all participants that what is said in the group should not be repeated outside of the group, we have no

Who can I talk to?

If you have questions, concerns, or complaints, or think the research has hurt you, talk to the research team at (305) 284-2002 with Dr. Guillermo “Willy” Prado, the person leading the research.

This research has been reviewed and approved by an Institutional Review Board (“IRB”). The Human Subject Research Office (HSRO) provides administrative support to the University of Miami’s IRBs. Please call the HSRO at 305-243-3195 if you are a participant in any research being conducted by UM, and:

- Your questions, concerns, or complaints are not being answered by the research team
- You cannot reach the research team
- You want to talk to someone besides the research team
- You have questions about your rights as a research subject
- You want to get information or provide input about this research

control over what happens outside of the group. You are reminded to not share anything you wouldn't want repeated outside of this group.

Your information may be looked at and/or copied for research or regulatory purposes.

A Certificate of Confidentiality (CoC) covers this research. A CoC helps protect your identifiable information.

A CoC protects your private information from all legal proceedings. Unless you assent, information from this research study that identifies you will not be shared outside this research except as described above.

- No one can be forced to share your identifiable information for a lawsuit.
- Your information can't be used as evidence even if there is a court subpoena.

The CoC does not prevent some things.

- The researchers can't refuse requests for information from those funding this research. The National Institute of Health may need information to assess this project.
- You can still share information about yourself. You can also freely discuss your involvement in this research, but this is your choice. The information you share will no longer be protected by the CoC.
- The researchers must disclose things required by law. This includes suspected child abuse and neglect, harm to self or others.

This study will be registered and may report results on www.ClinicalTrials.gov, a publicly available registry of clinical trials.

Payment

You will receive $20 for the first survey, $25 for the second, $30 for the third, and $35 for the last, for your time and effort.

PARTICIPANT'S STATEMENT/SIGNATURE

- *I have read this form and the research study has been explained to me.*
- *I have been given the chance to ask questions, and my questions have been answered. If I have more questions, I have been told who to call.*
- *I agree to be in the research study described above.*
- *I will receive a copy of this consent form after I sign it.*

____
Youth Name

____ ________________
Youth Signature Date

____
Printed Name of Person Obtaining Consent/Assent

____ ________________
Signature of Person Obtaining Consent/Assent Date

Future Studies

We may conduct additional studies related to the study we are describing in this form. If we do conduct these studies, we may contact you in the future to see if you would like to be part of the future study.

- I agree to be contacted after the study ends to be asked to take part in future studies.

____
Youth Name

____ ________________
Youth Signature Date

____
Printed Name of Person Obtaining Consent/Assent

____ ________________
Signature of Person Obtaining Consent/Assent Date
