# Supplementary material for: Familias con Orgullo: Study protocol for an efficacy study of a family-based intervention for Hispanic sexual minority youth
Source: PLoS One. 2023 Dec 15;18(12):e0295683. doi: 10.1371/journal.pone.0295683 (PMC10723681; doi:10.1371/journal.pone.0295683)
Supplement: S4 File — (DOCX) [file pone.0295683.s005.docx]

**Hair Cortisol Collection Procedures**

- 1. **Sample Collection and Storage**
     1. Label the outside of each tube and a plastic container with the following:
        1. Individual/patient name or identifying ID number.
        2. Date of Birth
        3. Date of Collection
        4. Time of Collection
        5. Initials of Collector
     2. Secure the entire length of hair to be sampled with a rubber band or clip.
     3. Cut hair as close to the scalp as possible (taking care not to nick the skin) with clean scissors.
     4. Place the hair sample in a pouch made of aluminum foil or a 15 ml screw-top polypropylene centrifuge tube.
        1. Note: Samples can be stored indefinitely at -20° C and, if necessary, shipped overnight at ambient temperature.
        2. Hair collection procedures should be practiced on volunteers, and the practice samples weighed. Hair samples as small as 5-10 mg can be analyzed; however, collecting samples > 10 mg is desired to minimize the likelihood of obtaining readings below the lowest CORT standard.
  2. **Transport to Biobehavioral Lab**
     1. Place the labeled bag in the refrigerator in the anteroom of room 340.
     2. Record sample information on the log on top of the refrigerator.
     3. Biobehavioral lab staff will retrieve samples and store them.
  3. **Sample Washing and Drying**
     1. Cut the hair sample into small pieces (1-3 mm)
     2. Weigh the 2 ml Eppendorf tube.
     3. Place into a 2 ml Eppendorf tube that is reinforced for bead beating.
     4. Reweigh the tube to obtain the hair weight.
     5. Record hair weight.
     6. Add 1.0 ml isopropanol to each tube.
     7. Place on the rotator for 10 minutes.
     8. Decant isopropanol.
     9. Add 1 .0 ml isopropanol to each tube.
     10. Place on the rotator for 5 minutes.
     11. Decant isopropanol.
     12. Place tubes into the speed vac evaporator to dry the solvent. (60 minutes – overnight).
     13. Weigh the tube with hair again and record and calculate net hair weight after wash**,**
  4. **Grinding**

As heat generated by the grinding process can cause damage to the sample, it is recommended that the samples be added to the tube containing the beads and stored for 12 hours at low temperatures (below -60), to achieve the best results.

- - 1. Place the sample into the prefilled grinding tube with zirconium beads.
    2. Remove the bead blaster rotor cover and place tubes in the rotor.
    3. Replace cover.
    4. Set speed, time, and cycle on the machine.
       1. Speed 6.5
       2. Cycle 3
       3. Time:30
       4. Intermission:30
    5. Grind hair into powder.
  1. Cortisol Extraction
     1. Add 1.0 ml methanol to each tube.
     2. Put the tube on a rotator with constant rotating for 24 hours.
     3. Centrifuge the tubes at 14,000 rpm for 5 minutes.
     4. Transfer 0.5 ml of the supernatant to a new 1.7 ml Eppendorf tube.
     5. Evaporate solvent – place in evaporator in concentrator for at least 90 minutes.

**Urine Drug Screen Collection Procedures**

##### Collect Urine Specimen

- - 1. Remove the cap from the urine cup.
    2. Donor provides urine specimen in the collection cup.
    3. Fill the cup to at least 1/3 full.
    4. Technician replaces and secures the cap while the cup is on a flat surface.
    5. Temperature is read at 2-4 minutes.
    6. Verify the range is between 90-100 ° F.
    7. The collector/technician dates and initials the security seal.
    8. Attach the security seal over the cup cap.
    9. Transport the cup to the biobehavioral lab.
    10. Record the sample drop off in the laboratory sample drop off binder located on top of the small black refrigerator located in the anteroom of 340.
  1. **Reading Results**
     1. Peel off the label to reveal drug test strips.
     2. Peel off label to reveal adulteration strips, if applicable.
        1. For cups with Adulteration
           1. Read adulteration test results between 2-5 minutes.
           2. Compare the colors on the adulteration strip to the color chart.
           3. If the results indicate adulteration, do not read the drug test results.
     3. Read the drug test results at 5 minutes. (Do not interpret results after 60 minutes, as false results may occur.
